# Comparative Single‐Cell Transcriptomic Atlas Reveals the Genetic Regulation of Reproductive Traits

**DOI:** 10.1002/advs.202517633

**Published:** 2026-01-21

**Authors:** Bingru Zhao, Hanpeng Luo, Dailu Guan, Xuefeng Fu, Feng Wang, Guoming Zhang, Shanglai Li, Hua Yang, Yanli Zhang

**Affiliations:** ^1^ Jiangsu Livestock Embryo Engineering Laboratory College of Animal Science and Technology Nanjing Agricultural University Nanjing China; ^2^ Department of Immunology School of Basic Medical Sciences Capital Medical University Beijing China; ^3^ Department of Animal Science University of California‐Davis Davis California USA; ^4^ Key Laboratory of Genetics Breeding and Reproduction of Xinjiang Wool‐Sheep Cashmere‐goat (XJYS1105) Institute of Animal Science Xinjiang Academy of Animal Sciences Urumqi China

**Keywords:** cross‐species comparisons, evolutionary conservation, GWAS integration, reproductive biology, single‐cell transcriptomics

## Abstract

Reproduction is a fundamental biological process regulated by complex cellular and molecular networks across the neuroendocrine and reproductive systems. To explore conserved and species‐specific mechanisms of fertility regulation, we constructed a high‐resolution single‐cell transcriptomic atlas of 15 reproductive and central nervous system (CNS) tissues from sheep and integrated it with human single‐cell datasets from 13 matched tissues. This comparative atlas comprises over 1.09 million cells and identifies 76 major cell types across species. Cross‐species integration based on 15 748 orthologous genes revealed that 54 cell types (71.1%) are shared between sheep and humans, showing strong conservation in transcriptional programs, cell lineage trajectories, and regulatory networks. Integrating genome‐wide association studies (GWAS) for sheep lifetime average litter size with the single‐cell atlas identified crucial fertility‐associated genes and signaling pathways. Cell–cell communication analysis revealed UNC5–SLIT–BMP signaling cascades coordinating neuroendocrine regulation of fertility along the hypothalamus–pituitary–ovary (HPO) axis. Trait–cell type enrichment analyses for 41 human complex traits further demonstrated that conserved reproductive and CNS cell types in sheep recapitulate key human GWAS associations. Together, this cross‐species single‐cell atlas (https://csca.njau.edu.cn/) provides a valuable resource for understanding how conserved cellular programs and inter‐organ signaling networks regulate fertility and other complex traits.

## Graphical abstract

A cross‐species single‐cell transcriptomic atlas of reproductive and central nervous system tissues from sheep and humans reveals conserved cellular programs and regulatory networks that regulated fertility. Integration with GWAS for sheep lifetime average litter size identifies UNC5–SLIT–BMP signaling as a core pathway coordinating neuroendocrine–ovarian communication, providing a valuable resource for dissecting the genetic regulation of reproduction.

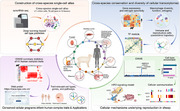

## 1 Introduction

Sheep (*Ovis aries*) is an important agricultural species, providing meat, milk, wool, and other products. Over the past decades, extensive research has been dedicated to understanding their genetics, physiology, and molecular mechanisms underlying agronomic traits in sheep [1, 2, 3, 4, 5]. According to the Sheep QTLdb (Aug 26, 2025), a total of 8,288 quantitative trait loci (QTLs) have been identified for a wide range of economic traits. Some of the well‐characterized causal genes were identified, such as *BMPR1B* for litter size [6], *RXFP2* for polledness [7], *HOXD1* for multi‐horn phenotype [8], and *PDGFD* for fat‐tail [9]. Meanwhile, considerable efforts have been made to elucidate the physiological, molecular, and cellular mechanisms underlying these traits. For example, a non‐conservative substitution (Q249R) at the *BMPR1B* locus has been shown to significantly increase ovulation rate, contributing to the hyperprolificacy phenotype [10, 11]. This gene is highly expressed in reproductive tissues such as the ovary, particularly during the follicular phase [12]. However, its upstream and downstream regulatory pathways at the single‐cell level remain unexplored, limiting our understanding of its cell‐type‐specific roles within heterogeneous reproductive tissues.

Reproductive performance is a central determinant of mammalian fertility, with far‐reaching implications for evolutionary biology and agricultural breeding programs [13, 14]. In domestic species such as sheep, improving reproductive performance remains a primary goal for genetic improvement to boost productivity and economic returns. In reproductive biology, sheep have been established as a large animal model [15, 16]. As polyovulatory mammals with substantial variation in prolificacy, they serve as a cornerstone species in global agriculture and a valuable complement to primates for studying fertility genetics and physiology [17]. Their larger body size compared with rodents allows precise ultrasound monitoring of ovarian follicular development, detailed examination of placental growth, and comprehensive profiling of circulating hormones and neurotransmitters [18, 19]. Despite their importance, reproductive traits are genetically and physiologically complex, often exhibiting low heritability and strong environmental influences. Physiologically, reproduction is governed by the coordinated activity of the hypothalamic–pituitary–gonadal axis, which integrates neuroendocrine and gonadal signals to regulate reproductive function [20, 21]. This physiological and genetic complexity highlights the need for high‐resolution approaches capable of resolving the cellular and molecular mechanisms underlying these traits.

Although sheep provide unique advantages as a reproductive model, the similarities at the cellular and molecular levels between sheep and human reproductive systems have not been comprehensively studied. In humans, thousands of genomic loci associated with reproductive traits and diseases have been identified through a genome‐wide association study (GWAS) approach [22, 23, 24, 25, 26]. For example, Venkatesh et al. [27] conducted GWAS meta‐analyses across seven cohorts in up to 42 629 cases and 740 619 controls and identified 25 genetic risk loci for male and female infertility. However, the extent to which the underlying molecular mechanisms of these loci are conserved or divergent between humans and sheep remains unclear, limiting the translational value of sheep in reproductive research. In our previous study, we used single‐cell RNA sequencing (scRNA‐seq) to profile developing ovaries and revealed conserved cell types, cellular composition, and gene expression networks between the two species [17]. Integrating ovine single‐cell profiles with human GWAS data not only recapitulated known candidate genes but also uncovered novel ones, particularly during developmental stages that lack human data. These findings demonstrate that scRNA‐seq is a powerful and high‐resolution approach for uncovering conserved molecular mechanisms and prioritizing candidate genes and therapeutic targets for reproductive disorders. Despite large‐scale projects such as Tabula Muris and the Human Cell Atlas [28, 29, 30], reproductive and neuroendocrine tissues remain underrepresented in current single‐cell resources, and comprehensive cross‐species comparisons are particularly lacking. This gap highlights the need for an atlas‐level, multi‐tissue single‐cell framework to systematically map conserved and species‐specific cellular programs underlying reproduction biology.

**FIGURE 1:**
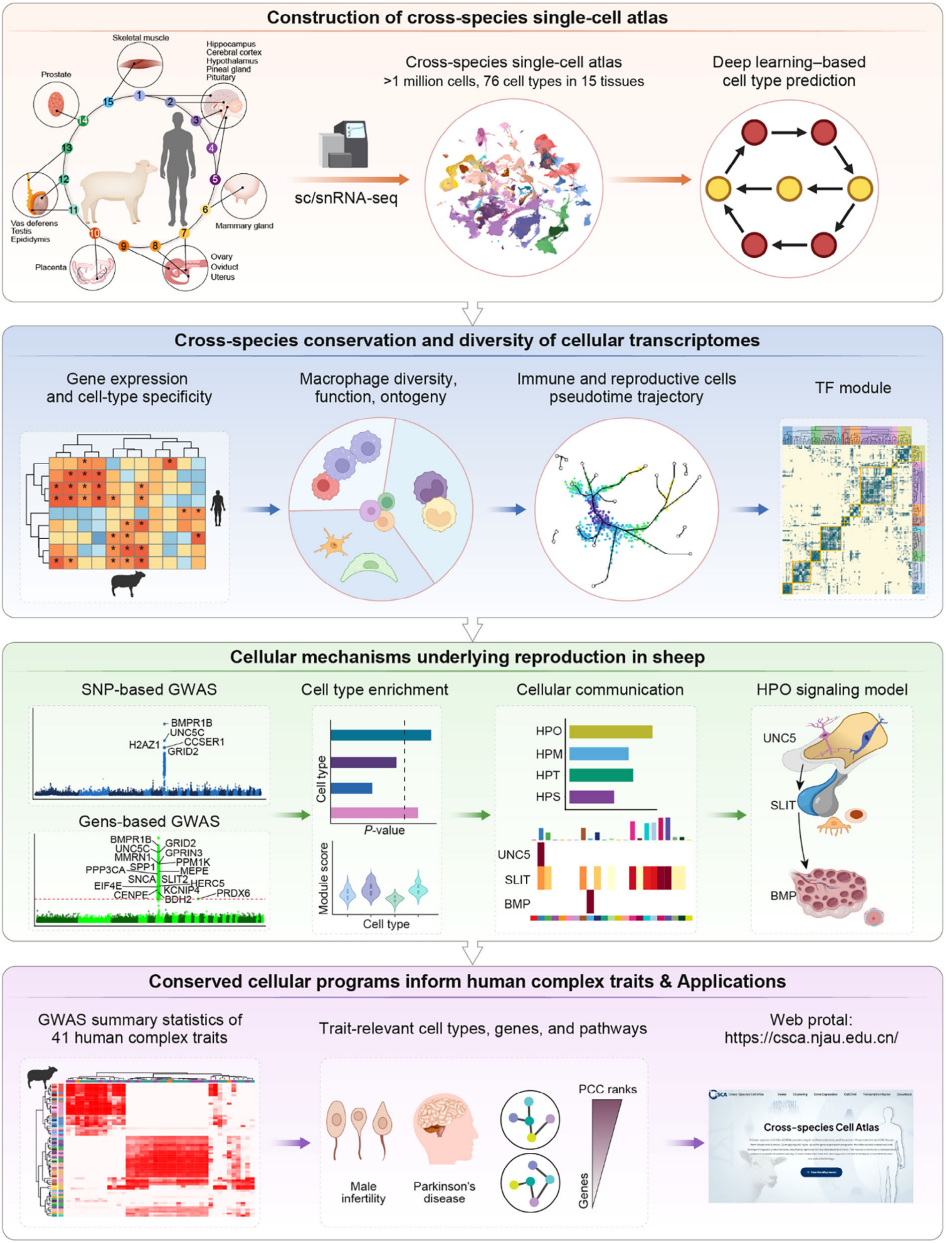
Schematic diagram of the study.

In this study, we aimed to perform an atlas‐level comparison of single‐cell transcriptomic profiles across reproductive and central nervous system (CNS) tissues between sheep and humans, with the overarching goal of elucidating how conserved and divergent gene expression programs influence reproductive traits (Figure 1). Specifically, we sought to address three key questions: (1) whether the same genes influence similar reproductive traits across species; (2) whether cellular architectures are conserved between sheep and humans; and (3) how sheep transcriptomic data can be leveraged to prioritize candidate pathways, genes, and potentially therapeutic targets for human reproductive diseases.

## 2 Results

### 2.1 Construction of a Comparative Single‐Cell Transcriptomic Atlas in Sheep and Humans

**FIGURE 2:**
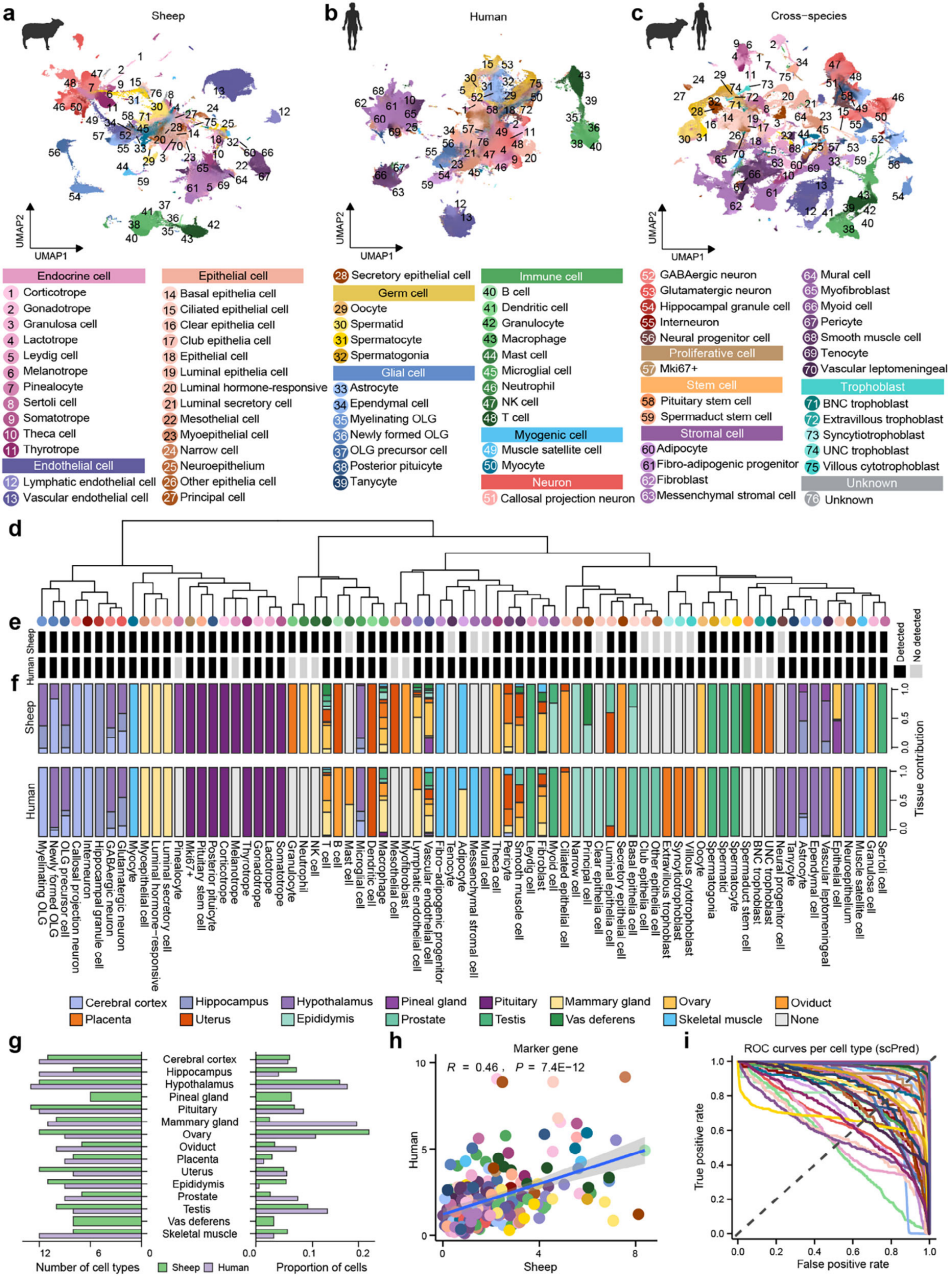
Construction of a cross‐species single‐cell atlas of sheep and human tissues. (a,b) Uniform Manifold Approximation and Projection (UMAP) plots showing the major cell types identified in sheep (a, 316 616 cells, 64 cell types) and humans (b, 769 901 cells, 65 cell types) single‐cell datasets. Cell‐type annotations are indicated by colors, consistently used throughout the manuscript. (c) UMAP visualization of integrated sheep and human transcriptomes based on 15 748 one‐to‐one orthologous genes, showing strong clustering by cell types across species (76 cell types in total). (d) Hierarchical clustering dendrogram illustrating transcriptional relationships among the 76 unique cell types from the integrated dataset. (e) Comparative cell‐type detection matrix between sheep and human datasets. Black squares indicate cell types detected; grey squares indicate undetected or absent cell types. (f) Bar charts showing the relative tissue contributions to each cell type in sheep (top) and humans (bottom). (g) Bar plots showing the number (left) and proportion (right) of high‐quality cells per tissue after quality control in sheep and humans. (h) Scatter plot showing the correlation of canonical marker gene expression used for cell‐type annotation between sheep and humans (Pearson's *r = *0.46, *P* = 7.4 × 10^−^^1^ ^2^). (i) Receiver operating characteristic (ROC) curve evaluating the performance of cross‐species cell‐type prediction using the scPred deep‐learning classifier trained on sheep data to predict human cell types. ROC curves approaching the upper‐right corner indicate high prediction accuracy. The *y*‐axis represents the true positive rate (sensitivity), and the *x*‐axis represents the false positive rate (1‐specificity).

To comprehensively investigate the cellular basis and mechanisms underlying reproductive biology, we generated a sheep single‐cell transcriptomic atlas encompassing 15 reproductive and CNS tissues, including cerebral cortex, hippocampus, hypothalamus, pineal gland, pituitary, mammary gland, ovary, oviduct, placenta, uterus, epididymis, prostate, testis, vas deferens, and skeletal muscle (Table S1). After rigorous quality control and cell‐type annotation, we obtained 316 616 high‐quality cells classified into 64 major cell types representing 12 distinct cell categories (Figure 2a; Figures S1a–d and S2a–c). To enable cross‐species comparison, we integrated publicly available human scRNA‐seq data from 13 matched tissues (pineal gland and vas deferens unavailable), obtaining 769 901 high‐quality human cells classified into 65 major cell types across 12 cell categories (Figure 2b; Figures S1e–h and S2d–f). Global multi‐lineage trajectories visualized using partition‐based graph abstraction (PAGA) [31] revealed the early emergence of tissue‐resident immune and stromal lineages in both species (Figure S2g,h).

Next, we integrated sheep and human datasets based on 15 748 one‐to‐one orthologous genes. We observed that the same cell types from the two species clustered together, indicating the global expression patterns were highly similar between sheep and humans (Figure 2c). To further investigate the cross‐species relationships among cell types, we performed an unsupervised hierarchical clustering analysis based on transcriptomic profiles across all 76 unique cell types (Figure 2d). Of these, 54 cell types (71.1%) were shared between the two species, indicating strong cross‐species conservation (Figure 2e). Notably, cell types clustered primarily by lineage rather than by tissue of origin, suggesting that transcriptional identity was driven more by cellular lineage than by anatomical location, and that neighboring cell types likely share conserved functional programs (Figure 2f). Both the proportion and the diversity of shared cell types within each tissue between species were conserved (two‐sided Student t‐test, *p* > 0.05), while species‐specific cell types were rare (<2.4%), likely reflecting low‐abundance populations that are difficult to detect (Figure S3). Therefore, downstream cross‐species comparison analyses focused on 54 conserved cell types and 15 748 one‐to‐one orthologous genes.

Among identified cell types, the number of cell types per tissue ranged from 6 (pineal gland) to 13 (pituitary) in sheep, and from 8 (testis) to 13 (hypothalamus) in humans, showing a strong inter‐species correlation (Figure 2g; Figures S4 and S5). Expression patterns of canonical marker genes used for cell‐type annotation exhibited high correlation between species (Pearson's *r* = 0.46, *P* = 7.4 × 10^−^^1^ ^2^; Figure 2h), exceeding the correlation observed when using the top 20 marker genes identified by Seurat (Pearson's *r *= 0.43, *P* < 2.2 × 10^−^^1^ ^6^; Figure S6 and Table S2). Cell cycle analyses further revealed conserved proliferative dynamics across both species and lineages. Germline and proliferative cells showed enrichment in the G2/M phase, indicative of active cell division, whereas stromal, endothelial, neuronal, and myogenic cells predominantly remained in the G1 phase, consistent with cellular quiescence (Figure S7).

To further assess transcriptional conservation between species, we applied the deep‐learning‐based classifier scPred [31] to predict 54 human cell types from the sheep dataset (excluding 11 rare cell types with fewer than 1000 cells). Receiver operating characteristic (ROC) curve analysis demonstrated high prediction performance, confirming strong conservation of core transcriptional identities across major cell types (Figure 2i). These results underscore the robustness of sheep‐to‐human cell‐type prediction and the reliability of cross‐species cell identity inference (Figures S8 and S9). Collectively, this high‐resolution cross‐species single‐cell atlas (CSCA) provides a valuable resource for comparative analyses in reproductive biology and is publicly accessible at https://csca.njau.edu.cn/.

### 2.2 Conservation and Diversity of Cellular Transcriptomic Profiles Across Species

**FIGURE 3:**
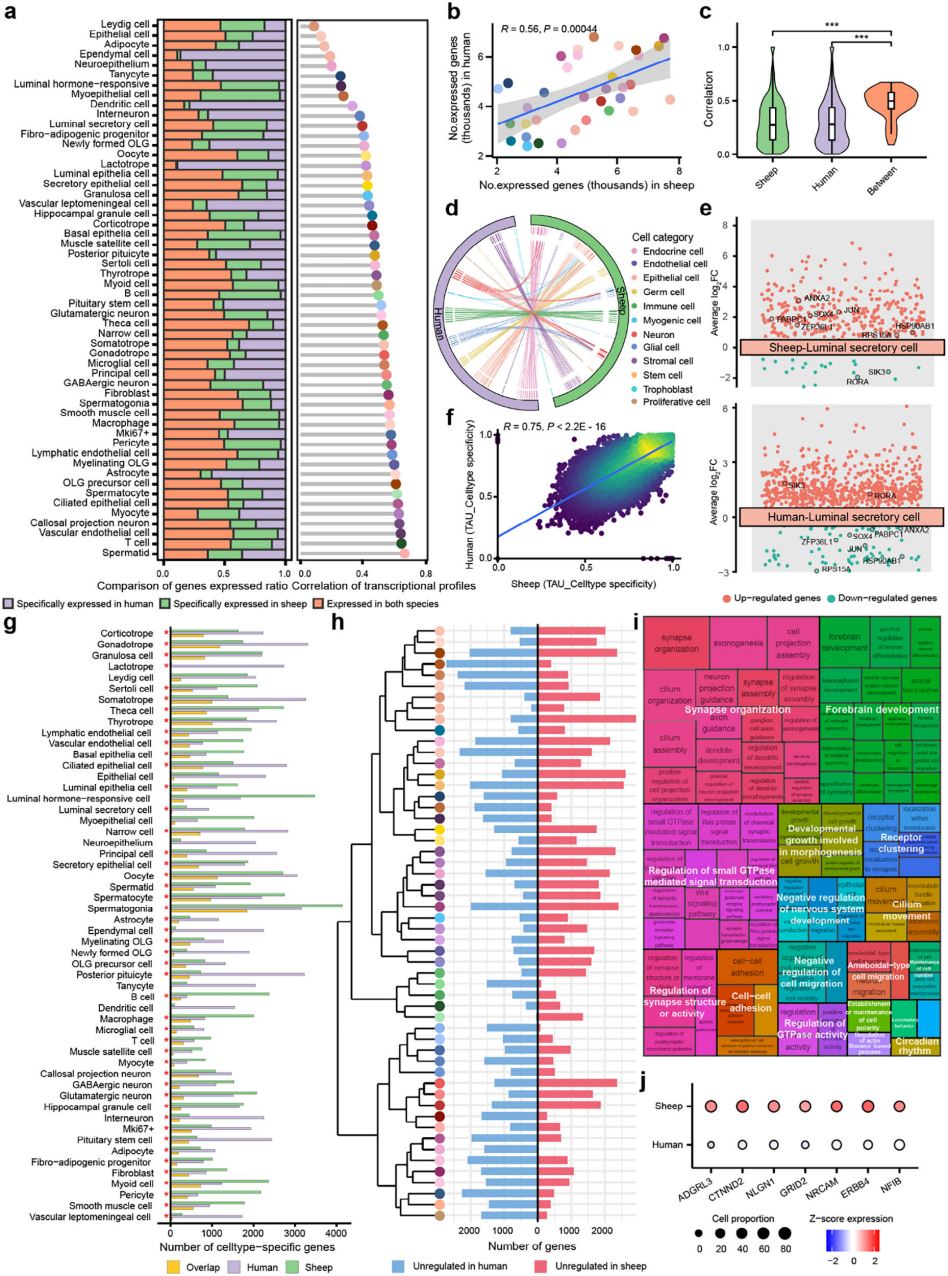
Comparison of cell landscapes across species. (a) Transcriptional similarity of cell types between sheep and humans. Left: proportion of orthologous genes expressed in both species or uniquely in one species. Right: Transcriptional similarity of corresponding cell types across species. (b) Pearson's correlation of the number of expressed genes per cell type between sheep and humans. Each dot represents a cell type. (c) Violin plots comparing gene expression correlations among cell types within sheep, within humans, and between corresponding cell types across species. “***” indicates significant differences (one‐sided Student's *t*‐test, FDR < 0.001). (d) Circos plot showing the similarity of cross‐species cell types. Lines connect cell type pairs with AUROC scores > .9. (e) Differentially expressed genes (DEGs) in luminal secretory cells between sheep and humans. (f) Correlation of cell‐type specificity (tau values) between sheep and humans. (g) Number of cell type‐specific genes identified in sheep and humans, and their overlapped genes across 54 conserved cell types. The overlapped genes were tested using a hypergeometric test. “*” represents FDR < 0.05 (Benjamini–Hochberg method corrected *p*‐value). (h) Hierarchical clustering of 54 conserved cell types based on integrated transcriptome data (left). Bar plot showing the number of species‐specific DEGs per cell type (right). (i) Gene ontology (GO) enrichment analysis of up‐regulated genes in sheep tanycytes compared to those of humans. (j) Expression of representative neuronal development‐related genes in tanycytes from sheep and humans.

To examine gene expression conservation across reproductive and CNS tissues, we systematically compared single‐cell transcriptomes from 54 conserved cell types in sheep and humans. On average, 44% of orthologous genes were expressed in the same cell types in both species (defined as detection in >25% of cells and expression levels exceeded the third quartile for the given cell type), although a subset of genes displayed species‐specific patterns (Figure 3a). The number of expressed genes per cell type was significantly correlated between both species (Pearson's *r* = 0.56, *P* = 4.4 × 10^−^^4^; Figure 3b). Notably, gene expression correlations between matching cell types across species were significantly higher than correlations between different cell types within each species (Figure 3c). Meta‐neighbor analysis further confirmed that similarities in orthologous gene expression were driven by cell type identity rather than species, particularly within neural and immune lineages (area under the receiver operating characteristics [AUROC] score > 0.9; Figure 3d; Figure S10). However, certain cell types, notably the epithelial lineage, exhibited accelerated evolutionary divergence between species (Figure 3e; Figure S11). For instance, luminal secretory cells in sheep showed species‐specific upregulation of genes involved in mitochondrial respiratory chain complex I, protein targeting to membranes, and neutrophil activation, reflecting potential adaptations in mitochondrial activity and secretory function [32], whereas these genes were downregulated or not expressed in humans (Figure 3e; Figure S12).

To assess the conservation of cell‐type specificity, we calculated tau values [33] as indicators of gene expression specificity across cell types. Tau values were highly and significantly correlated between sheep and humans (Pearson's *r* = 0.75, *P* < 2.2 × 10^−^^1^ ^6^; Figure 3f), supporting a conserved pattern of cell‐type‐specific expression. A similar pattern was observed for tissue‐specific expression (Figure S13a). Furthermore, we observed a significant negative correlation between phastCons scores (sequence conservation) and median absolute log_2_ fold changes (log_2_FC, humans compared to sheep) (Pearson's *r *= −0.26, *P* < 2.2 × 10^−^^1^ ^6^; Figure S13b), indicating genes with higher sequence conservation tend to exhibit lower expression divergence. Consistently, tau values were also negatively correlated with phastCons scores in both sheep (Pearson's *r* = −0.37) and humans (Pearson's *r* = −0.29; Figure S13c,d), suggesting that genes with highly cell‐type‐specific expression tend to be less conserved at the sequence level.

Cell type‐specific genes significantly overlapped between sheep and humans (hypergeometric test, FDR < 0.05; Figure 3g). These shared cell type‐specific genes generally displayed conserved expression patterns and biological functions across species (Figure S14). Cell types with similar cross‐species expression profiles shared a greater number of cell type‐specific genes (Figure S13g). Moreover, the degree of cell type‐specificity (measured by −log_10_ *P*) was positively correlated between species (Figure S13h), indicating that genes with higher cell‐type specificity tend to have more conserved expression patterns.

We next performed pairwise comparisons of gene expression across 54 conserved cell types between species (Figure 3h). Tanycytes exhibited the lower cross‐species correlation (Figure 3a), with 574 genes being significantly upregulated in sheep compared to humans (Figure 3h, Table S3). These genes were enriched for functions related to synapse organization, forebrain development, and nervous system regulation, such as *ADGRL3*, *CTNND2*, *NLGN1*, *GRID2*, *NRCAM*, *ERBB4*, and *NFIB* (Figure 3i,j). These findings suggest that sheep tanycytes may possess distinct transcriptional programs linked to neurodevelopmental processes and specialized forebrain functions. Together, these results demonstrate that despite some interspecies differences, the transcriptional programs and expression specificity of conserved cell types are largely maintained between sheep and humans.

### 2.3 Cellular Heterogeneity in Immune and Reproductive Lineages

**FIGURE 4:**
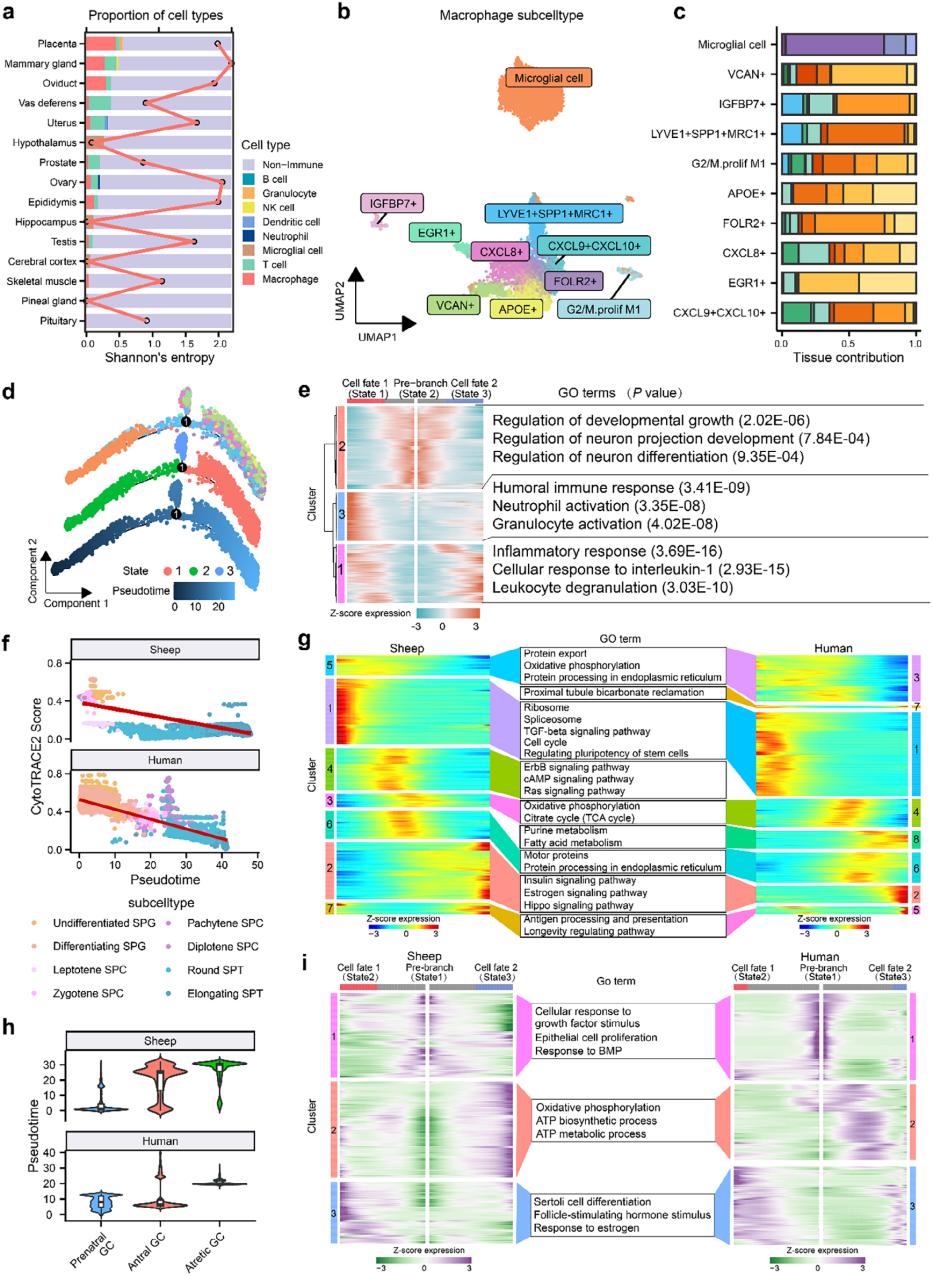
Cellular heterogeneity across immune and reproductive lineages. (a) Proportions of immune and non‐immune cell types across sheep tissues. The red line represents Shannon entropy values for immune cells in each tissue, reflecting the degree of immune cell diversity within each tissue. (b) UMAP visualization of macrophage subtypes in sheep. (c) Tissue contributions to each macrophage subtype in sheep. (d) Pseudotime trajectories of sheep macrophages, colored by modeled pseudotime (bottom), predicted cell states (middle), and subtypes (top). (e) Pseudo‐heatmap showing differentially expressed genes (DEGs) during the macrophage cell fate commitment (left), and top‐enriched Gene Ontology (GO) terms (*p* < 0.05) for each gene set (right) in sheep. (f) CytoTRACE2 scores decrease along pseudotime in germline cells from sheep and humans, consistent with reduced developmental potential during spermatogenesis. (g) Monocle2‐derived gene clusters from spermatogenic subtypes in sheep (left, 7 clusters) and humans (right, 8 clusters), with representative enriched GO terms for matched clusters. (h) Violin plots of pseudotime distributions across granulosa cell subtypes. (i) Heatmap showing gene clusters that vary along granulosa cell fate trajectories in sheep (left) and humans (right), with the top enriched GO terms (*p* < 0.05) for each cluster shown in the middle.

Given the broad distribution and functional diversity of immune cells across tissues, we first mapped the immune landscape to characterize tissue‐specific cellular heterogeneity. We profiled 26 735 immune cells from sheep and 59 820 from humans, classifying them into eight and six immune cell types, respectively (Figure 4a, Table S4, and Figure S15a,b). Reproductive tissues such as the ovary, oviduct, and mammary gland exhibited higher immune entropy (quantified as the Shannon diversity index of immune cell type proportions within each tissue) in both species (Figure 4a; Figure S15c), indicating conserved immune heterogeneity. Notably, macrophages were among the most widely distributed immune cell types, detected in 12 sheep tissues and 10 human tissues (Figure 4a; Figure S15c), making them an ideal population for examining cellular heterogeneity across tissue microenvironments. Using canonical marker genes [34, 35, 36], macrophages were further classified into 10 subtypes in each species, with five subtypes conserved across species, including microglial cells, VCAN^+^, IGFBP7^+^, LYVE1^+^SPP1^+^MRC1^+^, and G2/M proliferative M1‐like cells (Figure 4b; Figure S15d). Microglial cells were restricted to CNS tissues, including the cerebral cortex, hippocampus, hypothalamus, pineal gland, and pituitary (Figure 4c; Figure S15e), consistent with their conserved role as resident macrophages that mediate immune surveillance and maintain neuroimmune homeostasis [37].

Macrophage heterogeneity was reflected in their functional states, developmental origins, and tissue‐specific adaptations. Macrophages in reproductive tissues displayed M1‐like polarization in addition to a higher MHC‐II antigen‐presenting score (APS) that is common in CNS tissues, indicating specialized immune functions beyond classical immune privilege in reproductive tissues [38, 39] (Figure S16a–d and Table S5). Although antigen‐presenting ability varied among subtypes, MHC‐II APS was consistently higher than MHC‐I APS in both species (Figure S16e–h and Table S5). Moreover, VCAN^+^ and CD48^+^ subtypes expressed monocyte‐derived macrophage markers [35], whereas other subtypes expressed tissue‐resident macrophage markers [35], suggesting that distinct developmental origins contribute to the diversity of macrophage subtypes. Both subtype‐ and tissue‐specific transcriptional programs further underscore the complexity of macrophage functional states (Figure S17). Pseudotime analysis revealed a conserved bifurcated trajectory in both species, originating from microglia and branching into distinct terminal subtypes, marked by a shift from neurodevelopmental to immune activation programs (Figure 4d,e; Figure S15f,g).

Considering the central role of the testis in male reproduction, we next examined the heterogeneity and differentiation of spermatogenic cells. A total of 55 059 spermatogenic cells were profiled from sheep (*n* = 41 329) and humans (*n *= 13 730), including spermatogonia (SPG), spermatocytes (SPCs), and spermatids (SPTs), along with other subtypes (Figure S18a–d). Pseudotime analysis revealed a continuous developmental trajectory from SPG through SPCs to mature SPTs in both species (Figure S18e,f), consistent with previous findings in humans [37]. RNA velocity and PAGA [40] analysis further confirmed this directionality of differentiation. The gradual decline of CytoTRACE2 scores [41] pseudotime reflected the progressive loss of developmental potential during germ cell maturation (Figure 4f; Figure S18g–j).

Gene expression modules were strongly conserved between the two species, regulating fundamental processes such as stem cell maintenance, meiosis, and metabolism, while species‐specific modules were enriched for immune‐ and signaling‐related pathways (Figure 4g). Representative marker genes for SPG (DDX4, KIT, STRA8), SPCs (CCNB1, HORMAD1, SCML2), and SPTs (CA2, PGK2, TNP2) [42] exhibited similar expression dynamics along pseudotime (Figure S19). We further identified transcription factors (TFs) associated with distinct germline subtypes in both species, including conserved TFs such as E2F1 in undifferentiated SPG, TCFL5 in diplotene SPCs, SOX5 in round SPTs, and CREM in elongating SPTs, as well as species‐specific TFs like DMRT1 in sheep undifferentiated SPG, TRIM69 and OVOL1 in human round SPTs and diplotene SPCs (Figure S20). Collectively, these results reflected the conservation and divergence of spermatogenic transcriptional programs between sheep and humans.

To explore the heterogeneity of female reproductive cells, we analyzed granulosa cells (GCs) from ovarian tissues, which are essential for follicular development. In total, we profiled 15 085 GCs from sheep (*n* = 7267) and humans (*n *= 7818), and classified them into preantral, antral, and atretic subtypes (Figure S21a,b). Pseudotime analysis revealed a shared branchpoint where preantral GCs diverged toward antral or atretic subtypes, exhibiting similar developmental dynamics across species (Figure 4h; Figure S21c,d). At this branchpoint, three conserved gene clusters were identified in both species: cluster 1 enriched for growth factor and BMP responses; cluster 2 for oxidative phosphorylation and ATP metabolic processes; and cluster 3 for hormone and estrogen signaling (Figure 4i), indicating parallel molecular programs governing follicular development in sheep and humans.

### 2.4 Revealing the Critical Regulators of Cell Identity Across Species

**FIGURE 5:**
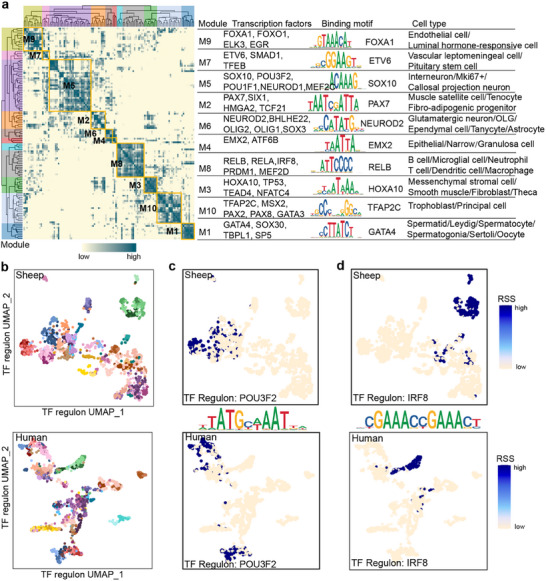
Cross‐species transcriptional network analysis of cell identity. (a) Identification of regulon modules based on the connection specificity index (CSI) matrix from the integrated single‐cell datasets of sheep and humans. The network comprises 119 orthologous transcription factor (TF) regulons grouped into 10 modules, each module annotated by representative TFs, their DNA‐binding motifs, and associated cell types. (b) UMAP visualization of all single cells based on regulon activity scores (RASs) for sheep (top) and human (bottom), with cells colored by major cell type. (c,d) UMAP visualization of RASs for representative lineage‐associated regulons for sheep (top) and human (bottom). (c) POU3F2 (Module 5, neuronal lineage). (d) IRF8 (Module 8, immune lineage).

To further dissect the transcriptional programs underlying cellular heterogeneity across tissues, we performed an integrated cross‐species network analysis to identify key transcriptional regulators of cell identity in sheep and humans. TF regulons were inferred using SCENIC [43] and their atlas‐wide similarity was assessed using the connection specificity index (CSI) [44]. In total, we identified 171 TF regulons in humans and 189 in sheep. Of these, 119 orthologous TF regulons were grouped into ten modules that were conserved in both species (Figure 5a, Table S6). For each module, we identified several representative regulators and cell types based on their average activity scores [45] (Figure 5a, Table S6). These modules were strongly associated with specific cell lineages, including germline (Module 1, M1), stromal (M3), epithelial (M4), neuronal (M6), immune (M8), and endothelial (M9) cells, and contained lineage‐defining TFs with conserved DNA‐binding motifs essential for lineage establishment and maintenance (Figure 5a).

Regulon activity scores (RAS)‐based distance clearly separated major cell types in both species, emphasizing conserved transcriptional regulation across species (Figure 5b). We further compared regulon specificity scores (RSS) among common, human‐specific, and sheep‐specific regulons. As expected, common regulons exhibited non‐significant RSS differences between species, consistent with their conserved regulatory roles. In contrast, human‐specific and sheep‐specific regulons displayed significantly higher RSS values within their respective species (*p* < 0.05), suggesting species‐specific regulatory activity shaping distinct transcriptional identities (Figure 5e). Species‐specific regulons were enriched for TF families such as basic helix–loop–helix (bHLH), Cys2–His2 zinc finger, homeodomain, and ETS (Table S6). Despite species‐specific differences, most cell lineage–specific regulons were conserved (Table S6). For instance, the POU3F2 regulon in neuron cells and the IRF8 regulon in immune cells were conserved between species (Figure 5c,d). POU3F2 is crucial for neuronal differentiation [46], whereas IRF8 regulates immune responses through interferon signaling [47], reinforcing the conserved functional roles of these transcriptional regulators.

### 2.5 Cellular Mechanisms Underlying Reproduction in Sheep

**FIGURE 6:**
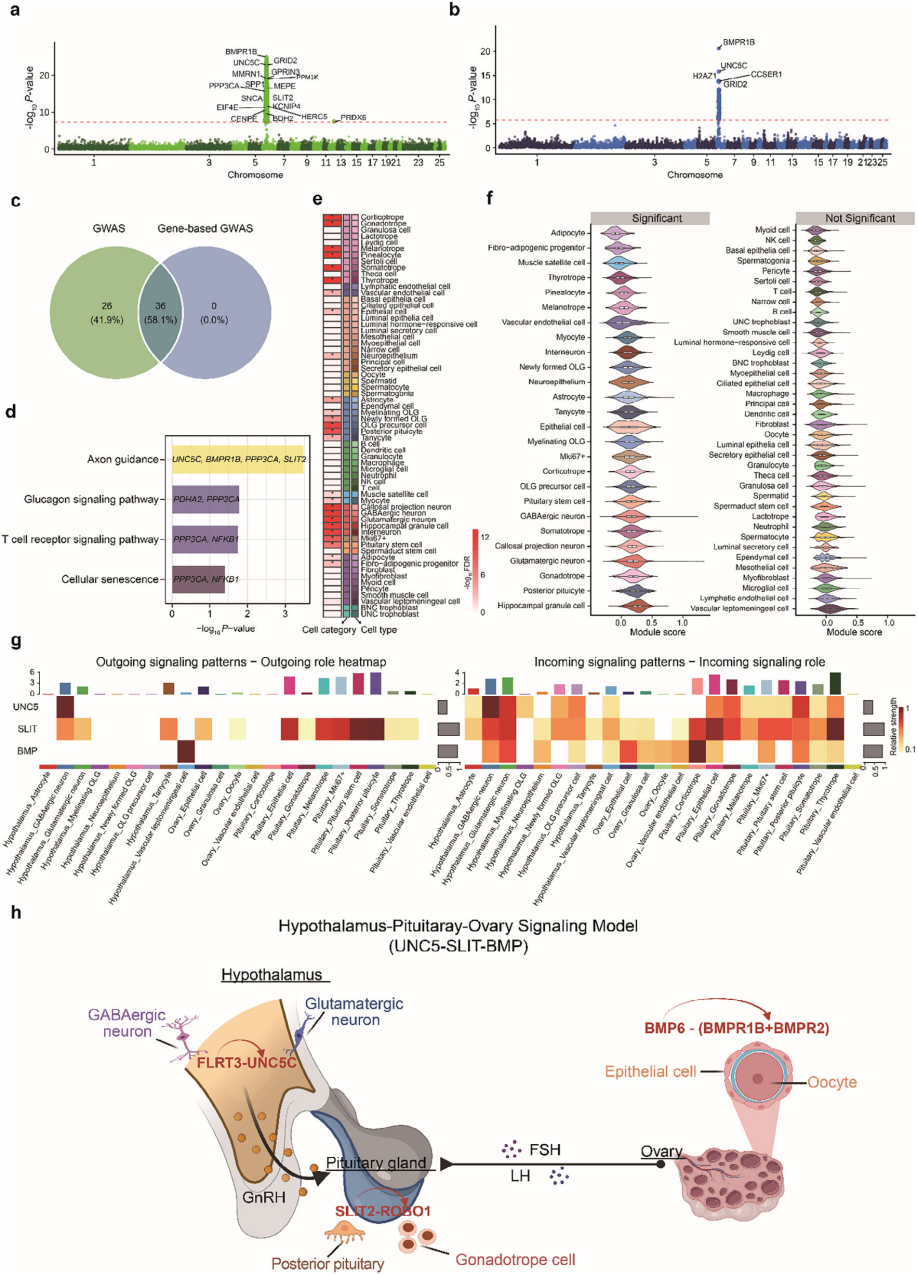
UNC5–SLIT–BMP signaling connects GWAS loci to cross‐tissue cell–cell communication along the hypothalamus–pituitary–ovary (HPO) axis. (a) Manhattan plot of SNP‐based GWAS for lifetime average litter size in sheep (*n* = 2180). The horizontal red line indicates the genome‐wide significance threshold. (b) Gene‐based association results using fastBAT based on cumulative SNP effects within gene boundaries. (c) Venn plot showing the overlap between SNP‐level and gene‐level associations. (d) KEGG pathway enrichment of overlapping genes, highlighting significant enrichment in axon guidance, BMP signaling, and cellular senescence pathways. (e) Associations (i.e., −log_10_FDR) between 65 cell types and the lifetime average litter size trait in sheep, as calculated using scPagwas. (f) Violin plots of module scores across cell types with fertility‐associated GWAS gene sets. (g) CellChat‐inferred intercellular communication within the HPO axis. (h) Proposed a cross‐tissue signaling model integrating FLRT3–UNC5C, SLIT2–ROBO1, and BMP6–BMPR1B/BMPR2 interactions.

To elucidate the cellular basis of sheep reproductive traits, we conducted GWAS for the lifetime average litter size (Figure S22) and integrated it with our sheep single‐cell atlas to identify cell types in which fertility‐associated variants act. We generated low‐coverage sequencing data (∼1×) and imputed to high coverage (∼20×), resulting in 1 152 006 high‐quality SNPs from 2,180 individuals after quality control (see Methods, Figure S22a,b). The statistical association identified 341 genome‐wide significant SNPs (Figure 6a). The further gene‐based mapping yielded 36 candidate genes (Figure 6b, Table S7), in consistent with SNP‐level associations (Figure 6c). These included the well‐established fertility‐related gene *BMPR1B* (i.e., *FecB*), and previously underappreciated candidates such as *UNC5C* and *SLIT2*, both of which are involved in neuronal signaling. Pathway analysis highlighted significant enrichment of neuron projection guidance and axon guidance genes (Figure 6d; Figure S22d), suggesting that neuroendocrine circuitry may play a role in reproductive regulation. The further fine‐mapping analyses identified several candidates with high‐confidence (PIP > 0.8) on chromosome 6, including *BMPR1B*, *UNC5C*, *SLIT2*, and *PPP3CA* (Figure S22e). As expected, the *BMPR1B* locus showed the strongest peak, consistent with its known effect on ovulation rate, while other loci highlighted additional neural‐related signaling pathways contributing to fertility.

To further delineate the cellular context in which these fertility‐associated genes exert their function, we performed trait–cell type enrichment analysis using scPagwas [48]. We observed strong enrichment in pituitary endocrine lineages, including corticotropes, thyrotropes, somatotropes, and gonadotropes, followed by hypothalamic neurons such as GABAergic and glutamatergic neurons (Figure 6e). Pathway‐level analysis confirmed that pituitary endocrine and GCs were not only enriched for fertility‐associated genes, but also enriched in GnRH secretion, oxytocin signaling pathway, axon guidance, and ovarian steroidogenesis (Figure S22f). To further validate these cell type‐specific enrichments, we calculated module scores using AddModuleScore based on the expression of GWAS gene sets. The modules were preferentially active in pituitary endocrine lineages and neuron lineages (Figure 6f). We observed a consistent pattern using AUCell [43] (Figure S23a), and the results from the two methods were highly correlated (Figure S23b), supporting the robustness of the observed cell‐type specificity.

To dissect how fertility‐associated signals are transmitted across tissues, we used CellChat [49] to model intercellular communication along four major tissue axes: hypothalamus–pituitary–ovary (HPO), hypothalamus–pituitary–testis (HPT), hypothalamus–pituitary–mammary gland (HPM), and hypothalamus–pituitary–skeletal muscle (HPS). The HPO axis exhibited the highest number and strength of inferred interactions (Figure S23c), indicating a highly connected and active signaling network. Within the HPO axis, UNC5, SLIT, and BMP were identified as dominant inter‐organ signaling pathways (Figure 6g). GABAergic neurons in the hypothalamus and posterior pituitary cells acted as the dominant signal senders, and ovarian epithelial cells were the major receivers (Figure 6g; Figure S23d). Ligand–receptor pairs (including FLRT3‐UNC5C, SLIT2‐ROBO1, and BMP6‐BMPR1B/BMPR2) were specifically enriched in corresponding sender‐receiver populations (Figure S23e–g). Hypothalamic neurons were the primary source of UNC5 and SLIT signaling, while BMP signaling was present in multiple cell types (Figure S23h).

Based on integrated analyses of GWAS, single‐cell transcriptomic data, and intercellular communication networks, we proposed a model in which UNC5–SLIT–BMP signaling cascades coordinate the neuroendocrine regulation of fertility along the HPO axis (Figure 6h). In this model, GABAergic neurons stimulate glutamatergic neurons through FLRT3–UNC5C signaling, thereby activating GnRH secretion. SLIT2–ROBO1 signaling connects the posterior pituitary to gonadotrope cells, promoting the release of FSH and LH. BMP6 derived from ovarian epithelial cells acts on oocytes through BMPR1B–BMPR2, facilitating follicular development and oocyte maturation. Together, these interactions form a cross‐tissue signaling circuit integrating neuronal, endocrine, and reproductive components. Notably, classical hormones such as GnRH, LH, and FSH are embedded within this cascade as downstream effectors, suggesting that neuroendocrine hormones may serve as executors of a broader intercellular communication network shaped by genetic regulation.

### 2.6 Conserved Cellular Programs Inform the Genetic Basis of Human Complex Traits

**FIGURE 7:**
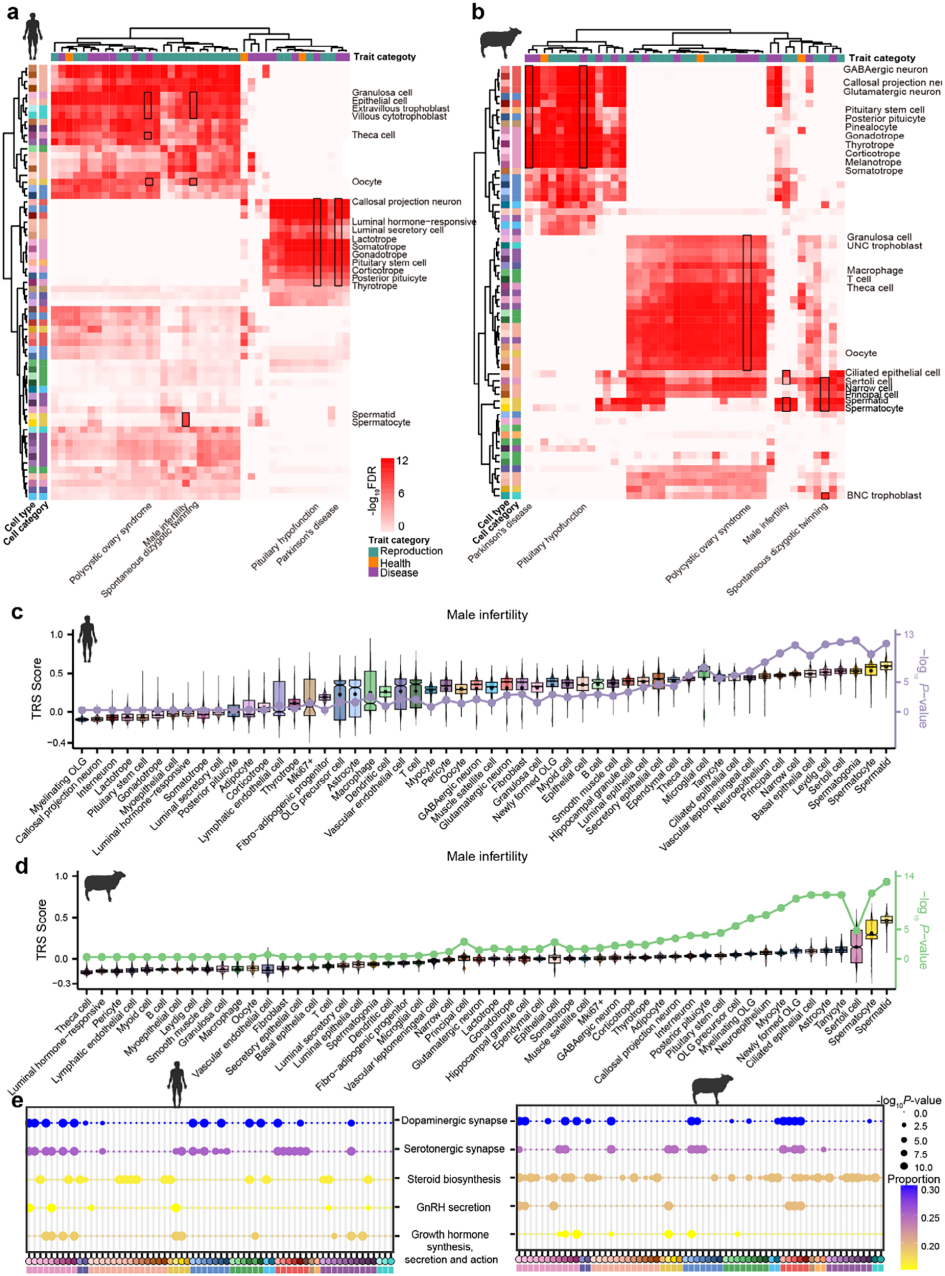
Cross‐species conservation of cell types and molecular pathways associated with human complex traits. (a,b) Heatmaps showing associations (−log_10_FDR) between conserved cell types and human complex traits based on trait‐cell type enrichment analysis using scPagwas in human (a) and sheep (b) single‐cell data. (c,d) Violin plots of trait‐relevant scores (TRS) for cell types associated with male infertility in human (c) and sheep (d). Dots indicate median TRS values, and lines indicate the significance levels (−log_10_ *P*‐value) of associations. (e) Dot plots showing conserved trait‐relevant pathways associated with male infertility in human (left) and sheep (right), identified by scPagwas. Dot size indicates the significance level (−log_10_ *P*‐value) for each pathway, and color intensity indicates the proportion of cells within each type influenced by these pathways (pathway‐level coefficient *β* > 0).

Given the strong conservation of cellular composition and gene expression patterns observed between sheep and humans, we next investigated whether sheep single‐cell data could provide insights into the genetic basis of human complex traits. We collected GWAS summary statistics for 41 human traits, representing reproduction (*n* = 23), health (*n* = 3), and disease (*n* = 15) (Table S8). To prioritize cell types associated with these complex traits, we performed trait‐cell type enrichment analyses using scPagwas [48] separately for sheep and humans, and identified certain associations between cell types and complex traits in both species (Figure 7a,b; Figure S22). These cell types showed high interspecies trait‐relevant scores (TRSs), indicative of conserved cellular functions between species. For instance, granulosa cells, theca cells, and oocytes were associated with polycystic ovary syndrome; trophoblasts with spontaneous dizygotic twinning; and neuronal lineages together with pituitary endocrine cells with pituitary hypofunction (Figure 7a,b).

Focusing on male infertility as a representative example, we observed higher TRSs and significant associations for spermatids, spermatocytes, and sertoli cells in the testis of both species (Figure 7c,d), emphasizing the conserved roles of germ cells in fertility regulation. Furthermore, we identified several critical trait‐relevant pathways, including dopaminergic synapse, serotonergic synapse, steroid biosynthesis, GnRH secretion, and growth hormone synthesis and secretion, consistently enriched in male infertility‐associated cell types (Figure 7e). These results suggest that these reproductive cell types contribute to the genetic architecture of male infertility through evolutionarily conserved biological pathways.

To further demonstrate the utility of sheep single‐cell data in predicting human neurological disorders, we investigated Parkinson's disease (PD) as an additional example (Figure S22). We found that callosal projection neurons in the cerebral cortex and specific endocrine cell types in the pituitary, including corticotropes, gonadotropes, somatotropes, and thyrotropes, showed significant associations and elevated TRSs for PD in both sheep and human datasets (Figure S22a,b). This indicates a conserved contribution of CNS cell types to disease etiology. Notably, several key genes such as RIMS2 [50], MAPK10 [51], and CACNA1C [52, 53] were consistently ranked among the top trait‐relevant genes in both species (Figure S22c,d). Pathway enrichment analysis further revealed that the top‐ranked trait‐associated pathways, such as GnRH signaling, ubiquitin‐mediated proteolysis, MAPK signaling, cortisol synthesis and secretion, and Parkinson's disease, were also conserved across species (Figure S22e,f). Together, these findings underscore the strong evolutionary conservation of reproductive and neurological cellular programs and demonstrate that cross‐species single‐cell analysis is a powerful framework for dissecting the genetic and cellular mechanisms underlying complex human traits.

## 3 Discussion

In this study, we constructed the first comprehensive multi‐tissue single‐cell transcriptomic atlas in sheep, providing a valuable resource for understanding cell‐type diversity and gene regulation across reproductive and CNS tissues. By profiling over 1.09 million cells from 15 ovine and 13 matched human tissues, we established a cross‐species framework to explore the cellular and molecular basis of mammalian reproduction. This large‐scale atlas not only expands upon previous bulk and tissue‐restricted ovine transcriptomic resources but also enables cell‐type–resolved comparisons between sheep and humans. Through integrative analysis, we found that cell types clustered primarily by lineage rather than tissue origin, revealing deeply conserved transcriptional programs that transcend anatomical boundaries [54, 55]. More than 70% of identified cell types were shared between species, with strong interspecies correlations in marker gene expression, cell cycle dynamics, and predicted cell identities, reinforcing the notion that core cellular phenotypes are conserved across mammalian evolution [56, 57].

Our atlas further delineated fine‐grained heterogeneity within macrophage, spermatogenic, and GC populations. Macrophages displayed a conserved bifurcated developmental trajectory separating tissue‐resident and monocyte‐derived lineages, consistent with previous findings in murine and human studies [58, 59]. Macrophages in reproductive tissues exhibited high MHC‐II–mediated antigen‐presenting capacity, consistent with recent reports that ovarian macrophages contribute to follicular development and ovulatory processes [60]. Spermatogenic trajectories from undifferentiated SPG to mature SPTs were conserved, sharing stage‐specific TFs such as E2F1 and CREM, while species‐specific TF like DMRT1 in sheep SPG highlighted evolutionary divergence in germ cell regulation. Given their essential role in folliculogenesis and endocrine regulation, granulosa cells exhibited a conserved pseudotime bifurcation into antral and atretic fates, consistent with human folliculogenesis [61, 62]. Regulon analysis identified ten conserved TF modules aligned with major cellular lineages, including POU3F2 for neuronal identity and IRF8 for immune function. These results align with large‐scale regulatory network studies showing that core TF regulons are deeply conserved across vertebrate evolution, while species‐specific regulons enriched for bHLH, zinc finger, and ETS families may underpin adaptive diversification in reproductive and neuroendocrine systems [63].

Integration of GWAS results for lifetime average litter size in sheep with our single‐cell atlas identified pituitary endocrine and ovarian follicular cells as key cellular contexts for genes associated with reproductive traits, consistent with the established roles of gonadotropes and GCs in ovulatory regulation [17, 64]. Among the top candidates, *BMPR1B*, a well‐known fecundity gene in sheep, modulates follicular development through the BMP/SMAD signaling pathway and directly influences ovulation rate [10, 11]. Notably, *UNC5C* has been found to associate with litter size in Hu sheep [60, 61] and conception rate in cattle [62]. Meanwhile, *SLIT2*, traditionally associated with axon guidance, also involved in epithelial cell differentiation, fertility regulation, and ovulation [65]. Moreover, our cell–cell communication analysis further revealed UNC5–SLIT–BMP signaling cascades along the HPO axis, representing a novel pathway in neuroendocrine regulation of reproduction. Although these findings suggest potential candidate genes in fertility regulation, further functional studies are required to validate their causal effects.

We further performed trait–cell type enrichment analyses for 41 human complex traits and found that conserved reproductive and CNS cell types in sheep recapitulate known human GWAS associations. For instance, GCs were associated with polycystic ovary syndrome, trophoblasts with dizygotic twinning, and pituitary endocrine cells with hypopituitarism. Moreover, the strong correspondence in trait‐relevant pathways, including GnRH, steroid biosynthesis, and dopaminergic signaling, highlights the translational potential of large‐animal single‐cell data for interpreting human complex traits [66]. Compared with rodent models, large animals such as sheep and cattle share greater physiological and endocrine similarity with humans, allowing cross‐species validation of GWAS findings in comparable cellular contexts [17, 34]. Their accessibility to reproductive and neuroendocrine tissues also facilitates in‐depth mechanistic and longitudinal studies that are not feasible in humans due to ethical and technical constraints. Collectively, these advantages position large‐animal single‐cell atlases as a critical bridge between human genomics and experimental validation, complementing existing primate and rodent models. Notably, similar cross‐species approaches have successfully bridged human and non‐human primate atlases in neurological disease research [22], underscoring the broader potential of comparative single‐cell resources.

Together, the CSCA provides a foundational resource for understanding how conserved and species‐specific cellular programs shape reproductive function across mammals. Future work integrating this atlas with functional genomics and genome editing approaches, such as CRISPR‐based perturbations in primary ovary or pituitary cells, will be essential for establishing causal relationships between candidate genes, cell states, and reproductive outcomes. These efforts will not only validate the mechanistic hypotheses generated from this atlas but also accelerate the translation of cross‐species insights into targeted interventions for improving fertility and reproductive health.

## 4 Conclusion

Overall, we present a large‐scale cross‐species single‐cell atlas of reproductive and CNS tissues in sheep and humans, revealing extensive conservation in cellular composition, transcriptional programs, and regulatory networks. By integrating GWAS with single‐cell data, we link the loci associated with reproductive traits to specific neuroendocrine and germ cell types, and reveal conserved inter‐organ signaling circuits along the HPO axis. Our findings highlight axon guidance pathways as previously underappreciated regulators of mammalian fertility and demonstrate the value of sheep as a translational model for human reproductive and neurological traits. The CSCA resource (https://csca.njau.edu.cn/) provides a foundational framework for dissecting the genetic and cellular basis of complex traits across species and contributes to the future development of precision breeding and therapeutic strategies.

## 5 Experimental Section

### 5.1 Ethics Statement

The animal procedures were approved by the Animal Ethical and Welfare Committee of Nanjing Agriculture University, China (Approval No. NJ202306002). All applicable institutional and national guidelines for the care and use of animals were followed, and all efforts were made to minimize animal suffering.

### 5.2 Sample Collection

Hu sheep, a domesticated Chinese breed renowned for its year‐round estrus and exceptionally high fecundity, were selected as a model species for reproductive biology. A total of seven healthy Hu sheep (five estrous ewes and two rams) were obtained from the National Hu Sheep Breeding Farm (Jiangsu, China). After a 12‐hour fast, animals were euthanized, and 15 tissues were collected across four physiological systems: CNS, female reproductive, male reproductive, and skeletal. Fresh tissue was pre‐rinsed with 1 × PBS precooling to remove residual blood and debris, and then cut into small pieces (∼0.5 × 0.5 cm) on ice using sterile scissors. Samples from the mammary gland, ovary, oviduct, placenta, uterus, epididymis, prostate, testis, and vas deferens were stored at 4°C in MACS Tissue Storage Solution (Miltenyi Biotech, 130‐100‐008) and protected from light until dissociation for scRNA‐seq. Additionally, samples from the cerebral cortex, hippocampus, hypothalamus, pineal gland, pituitary, and skeletal muscle were placed in cryogenic vials, snap‐frozen in liquid nitrogen, and stored at −80°C until nuclear extraction for snRNA‐seq.

To expand tissue coverage, we incorporated seven publicly available single‐cell or single‐nucleus transcriptomic datasets, including six ovarian samples [67] and one testicular sample [68] from healthy Hu sheep. In total, the sheep single‐cell transcriptome dataset comprised 35 samples representing 15 tissues (Table S1). For interspecies analysis, we further integrated human single‐cell transcriptome data from 170 samples across 13 tissues (Table S1).

### 5.3 Preparation of Single‐Cell Suspensions

Tissues were washed three times with 1× PBS, minced on ice, and digested with an Enzymatic digestion solution appropriate for each tissue type. The digested cells were filtered through a 40 µm nylon strainer (BD Falcon), centrifuged by centrifugation (500 rpm, 10 min, 4°C), and resuspended in base solution containing 0.2% FBS (Gibco). Cells were manually counted in triplicate by Trypan blue exclusion after each centrifugation and resuspended at a concentration of ≥2 × 10^6^/mL, and the cell viability exceeded 85%. Single‐cell suspensions were loaded onto the Chromium Controller (10× Genomics) according to the manufacturer's protocol.

### 5.4 Preparation of Single‐Nucleus Suspensions

Single nuclei were isolated using the 10× Genomics Isolation of Nuclei for Single Cell RNA Sequencing kit. Briefly, 1 mL pre‐chilled LB buffer was added to minced frozen tissue in a 2 mL Eppendorf tube, incubated on ice (1–10 min), and triturated. Lysates were filtered through a 40 µm SmartStrainer, centrifuged (500 × g, 5 min, 4°C), and resuspended in 300 µL LB buffer. The nuclei were mixed with 300 µL RB buffer, after which 600 µL PB1 and 600 µL PB2 buffers were sequentially underlaid at the bottom of the tube. Following centrifugation (4000 × g, 20 min, 4°C), the nuclei layer (150–300 µL) at the PB1 and PB2 interface was carefully aspirated and mixed with 1 mL RB buffer. The suspension was filtered again through a 40 µm SmartStrainer, centrifuged (500 × g, 5 min, 4°C), and finally resuspended in 100 µL EB buffer.

### 5.5 ScRNA‐Seq Library Preparation and Sequencing by 10×Genomics Platform

A total of 28 single‐cell or single‐nucleus RNA‐seq libraries were constructed from the newly collected sheep tissues using the Chromium Single Cell 3’ Library and Gel Bead Kit (10× Genomics), following the manufacturer's protocol. Gel Beads in Emulsion (GEMs) were generated using the Chromium Controller to enable cell lysis and barcoded reverse transcription of mRNA within individual droplets. Libraries were sequenced on the Illumina NovaSeq 6000 platform with 150 bp paired‐end reads.

### 5.6 Data Processing and Analysis of ScRNA‐Seq Data

Raw sequencing data were processed for demultiplexing, alignment, and read counting using CellRanger (v7.2.0). For the sheep samples, reads were mapped to the sheep reference genome (GCF_016772045.1_ARS‐UI_Ramb_v2.0). For the human samples, reads were mapped to the human reference genome GRCh38 (hg38). Gene expression matrices were further processed in Seurat [69] (v5.1.0) to remove low‐quality cells and genes. Cells were retained if they expressed 200–6000 genes, a percentage of mitochondrial genes < 10%, decontX [70] <0.9, and log_10_GenesPerUMI > 0.8; genes were kept if they expressed in ≥3 cells. Potential doublets were identified and removed using DoubletFinder [71] (v2.0.2).

After quality‐control, expression values were normalized using the function of “LogNormalize” (scale factor = 10 000). Highly variable genes (*n* = 2000) were identified using functions of “FindVariableFeatures” (method = “vst”) and scaled with “ScaleData”. Dimensionality reduction was performed by principal component analysis (PCA), with the optimal number of PCs determined by the “permutationPA” function in the jackstraw R package (https://github.com/ncchung/jackstraw). Batch effects across multiple samples were corrected using Harmony [72] (v1.2.0). Cell clustering was performed with the “FindClusters” function (resolution = 0.5) and visualized using the “RunTSNE” and “RunUMAP” functions.

### 5.7 Identification of Cell Types and Differentially Expressed Genes

Cell types for each cluster were annotated using three complementary strategies: (1) Clusters were manually annotated to specific cell types or subtypes based on the expression of well‐established marker genes reported in previous studies. (2) Automated annotation using the ScType [73] R package. (93) Positive marker genes for each cluster were identified with the “FindAllMarkers” function in Seurat, using thresholds of adjusted *p*‐value < 0.05, log_2_ fold change > 0.25, and detection in at least 25% of cells within the cluster.

Differentially expressed genes (DEGs) between each cell group and other groups were identified using the “presto:::wilcoxauc.Seurat” function with the thresholds of adjusted *p*‐value < 0.05 and |log_2_foldchange| > 0.25. Functional enrichment of DEGs for Gene Ontology (GO) and Kyoto Encyclopedia of Genes and Genomes (KEGG) pathways was conducted by using the ClusterProfiler [74] R package.

### 5.8 Integrative Analysis of Cross‐Species Single‐Cell Transcriptomic Datasets

For cross‐species integration, the combined expression matrices from each species were used as input. We used the same Seurat integration pipelines to integrate the datasets from sheep and humans. Only one‐to‐one orthologous genes between the two species, identified through Ensembl BioMart, were retained for downstream analysis. Batch effects across datasets were corrected using Harmony [72] (v1.2.0). The cluster distance matrix was computed using Spearman correlation and was then used for hierarchical clustering with the Ward.D2 method to generate dendrograms.

### 5.9 Cell Cycle Index Estimation

Cell cycle state was evaluated using the “CellCycleScoring” function in Seurat [69], which categorized each cell into the G1, S, or G2/M phases based on canonical marker gene expression [75]. Cells with the highest score less than 0.3 were classified as non‐cycling [76].

### 5.10 RNA Velocity Analysis

To model transcriptional dynamics, RNA velocity was estimated using sdevelo [77] (v0.2.12), a stochastic differential equation–based deep learning framework. Spliced and unspliced transcript counts were extracted from alignment files using velocyto [78] (v0.17.17).

### 5.11 Predicting Cellular Potency

The developmental potential of individual cells was inferred using CytoTRACE2 [41] (v1.0.0), which estimates cellular plasticity based on transcriptional diversity. Normalized gene expression matrices were analyzed with default parameters to generate a CytoTRACE score for each cell, where higher scores indicate greater transcriptional entropy and stemness potential. Analyses were performed separately for each sample, and the resulting scores were aggregated to enable cross‐cluster comparisons of inferred potency states.

### 5.12 Gene Regulatory Network Analysis

Gene regulatory networks (GRNs) were reconstructed using the SCENIC pipeline [43]. Raw expression matrices (log‐normalized) were filtered to retain genes with sufficient expression across cells, and TFs were identified based on organism‐specific databases. Co‐expression networks were inferred with GRNBoost2 in pySCENIC (v0.12.1), generating TF–target candidate links. Cis‐regulatory motif enrichment was performed using RcisTarget with the human motif database, including upstream and downstream regions surrounding transcription start sites. TF–target modules (regulons) were identified by the intersection of co‐expression modules and motif enrichment results. Regulon activity scores (RASs) were calculated for each cell using the AUCell [43] R package, yielding a regulon‐by‐cell matrix representing TF activity across cell types. The RAS was measured as the area under the recovery curve. The activities associated with each cell type were evaluated by calculating the RSS [45].

### 5.13 Pseudotime Trajectory Analysis

Cell lineage trajectory was inferred by Monocle [79] (v2.20.0). Cells were embedded into a two‐dimensional space, ordered along pseudotime, and visualized as a tree‐like structure with multiple branches representing divergent developmental paths.

### 5.14 Regulon Module Analysis

Regulon modules were identified based on the CSI [44], which is a context‐dependent measure for identifying specific associating partners. Hierarchical clustering with Euclidean distance was performed based on the CSI matrix to identify distinct regulon modules. A CSI > 0.7 as a threshold was used to construct the regulon association network to investigate the relationship of different regulons. For each module, its cell‐type‐specific activity score was calculated as the mean activity of all regulons in that module across cells of a given type, and the highest‐scoring cell types were identified for each module.

### 5.15 Cellular Communication Analysis

To identify and visualize cellular cross‐talk between cell types, the CellChat [49] package (v1.6.1) was used to infer ligand‐receptor pairs with default parameters. For sheep datasets, gene symbols were converted to human orthologs prior to analysis. Over‐expressed ligands and receptors were identified by “identifyOverExpressedGenes” and “identifyOverExpressedInteractions” functions. The “computeCommunProb” and “filterCommunication” functions were used to compute communication probability and infer cellular communication networks for each ligand‐receptor pair and signaling pathway. Pathway‐level communication networks, including interaction frequency and strength between cell types, were inferred using the “computeCommunProbPathway” and “aggregateNet” functions. Ligand–receptor pairs with *p*‐value < 0.05 were considered significant.

### 5.16 Cell Type Conservation Analysis

To attenuate the effects of noise and outliers, we generated pseudo‐cells [80] by averaging 30 randomly selected cells from the same cell type. Cross‐species transcriptional similarity was assessed using MetaNeighbor [81] (v1.18), with the “MetaNeighborUS” function calculating Spearman correlations between cell types based on AUROC scores. PhastCons [82] conservation scores calculated on multiple sequence alignment of sequences of 100 species of vertebrates were retrieved using the UCSC table browser data retrieval tool [83] (https://hgdownload.soe.ucsc.edu/goldenPath/hg38/).

### 5.17 Cell Type Diversity Analysis

The Shannon entropy was calculated to evaluate cell type diversity in each tissue according to the formula −∑_i_(*p*_i_ × log2(*p*_i_)), where *p*_i_ is the proportion of cell type in cell class i for each tissue.

### 5.18 Functional Scoring of Macrophage States

To evaluate macrophage polarization and antigen presentation abilities, the “AddModuleScore” function in the Seurat package was used to calculate the APS and macrophage_type score. The classical gene sets were from published literature (Table S5). Statistical significance was calculated using a two‐sided unpaired Student's *t*‐test, with *, **, ***, and **** indicating *p*‐value < 0.05, 0.01, 0.001, and 0.0001, respectively.

### 5.19 Genotype Imputation and Quality Control

Whole‐genome sequencing (WGS) was performed on genomic DNA extracted from peripheral blood using the Illumina NovaSeq 6000 platform. We performed joint variant calling with sequencing data for a reference population of 542 ewes. Alignment and variant calling of the DNA sequencing read followed the Genome Analysis Toolkit (GATK) best practice guidelines [84]. Default parameters were used for bwa‐mem [85] and sambamba [86] to align reads and to mark PCR duplicates, respectively. Single‐nucleotide variants and small indels were called with GATK HaplotypeCaller. Raw SNPs were subjected to strict variant‐level quality control, following established hard‐filtering recommendations for short‐read WGS data. Specifically, variants were removed if they exhibited evidence of poor mapping quality (MQ < 40), strand bias (FS > 60 or SOR > 3), low variant confidence normalized by depth (QD < 2.0), or significant mapping/positional bias based on read‐level annotations (MQRankSum < –12.5 or ReadPosRankSum < –8.0). We applied Beagle (v5.1) to phase the genotype. To improve variant resolution, genotype imputation was performed in two steps. First, a reference panel comprising 542 deeply sequenced individuals was phased using Beagle 5.4, generating a high‐quality haplotype reference with a 15 cm window size and 0.5 cm overlap, which improves local haplotype reconstruction in regions of elevated recombination while maintaining computational efficiency. The phasing procedure was run with 10 burn‐in iterations and 20 sampling iterations. The effective population size was set to 1000, and the genotype error rate was set to 0.0001. Next, genotype imputation of low‐coverage samples (*n* = 2180) was conducted using GLIMPSE2 [87] (v2.0.0), a reference‐based method optimized for low‐depth WGS data. The genome was divided into 1 Mb windows with 200 kb overlaps, and imputation was run with a minimum mapping quality of 20 and an effective population size of 1000. Initial variant‐level quality control was performed using PLINK [88] (v 1.9.0). Variants were retained if they met the following criteria: minor allele frequency (MAF) > 0.05 and Hardy‐Weinberg equilibrium (HWE) *p* > 1 × 10^−^^6^. Linkage disequilibrium (LD) pruning was conducted using a sliding window of 50 SNPs, with a step size of 10 SNPs and a pairwise *r* ^2^ threshold of 0.2 (–indep‐pairwise 50 10 0.2). The final pruned SNP set was used for downstream association testing.

### 5.20 GWAS and Fine‐Mapping

We performed the GWAS for the reproduction trait (lifetime average litter size) using GEMMA [89] (v0.98.3) in a population of 2180 sheep with 1 152 006 high‐quality SNPs. Fixed Covariates included farm and year effects. We considered Bonferroni 0.05 as a threshold to identify the significant SNPs (*P *< 4 × 10^−^^8^). We identified the causal variants linked to the significant SNPs by analyzing the summary statistics of GWAS using the susie_rss function in the susieR [90] package (v0.12.35), providing the SNP effect sizes, standard errors, and the LD matrix computed from the study population as input. The fine‐mapping model was run using *L* = 10 as the maximum number of causal components. Subsequently, the causal effect was evaluated by computing PIP. Locus plots with gene annotations were plotted using the locuszoomr R package (v0.3.5).

### 5.21 Gene‐Based Analysis

Gene‐based analysis was performed using fastBAT analysis as implemented in GCTA [91] (v1.94.1). fastBAT performs a set‐based association analysis, identifying genes enriched for nearby risk variants. Genes significantly associated with the trait (*P* < 1.7 × 10^−^^6^). Genes were identified by their name and genomic location.

### 5.22 Calculation of Module Scores

To assess the robustness of gene set activity estimation, we compared the AddModuleScore function from Seurat and AUCell [43] (v 1.30.1) for module scores across cell types with fertility‐associated GWAS gene sets. For both methods, we computed cell‐wise module scores across all annotated cell types and then aggregated scores by cell identity. Spearman's correlation was used to compare the consistency of module scores between the two methods. The high concordance supported the robustness of cell‐type‐specific enrichment results.

### 5.23 Enrichment Analysis Between Cell Types and Complex Traits

scPagwas [48] (v1.3.0) was a pathway‐based polygenic regression method that integrates scRNA‐seq data and GWAS data to identify cell populations critical for complex diseases and traits. To prioritize the top trait‐associated genes, we used the “scGet_PCC” function, which ranks genes based on the Pearson correlation coefficient (PCC), the expression of each gene, and the summed genetically associated pathway activity score across all cells. The “scPagwas_perform_score” function was then applied to analyze pathway activity within each cell type and determine the significance of active pathways using the singular value decomposition method.

## Author Contributions

B.Z. and H.L. contributed equally to this work. Conceptualization was done by Y.Z. and B.Z. Investigation was done by B.Z. and H.Y. Formal analysis, methodology and visualization were done by B.Z., H.L., H.Y., and S.L. Resources were done by Y.Z., X.F., and F.W. Supervision was done by B.Z., D.G., F.W., and Y.Z. Original draft was written by B.Z. Review Writing and editing were done by B.Z., H.L., D.G., and Y.Z.

## Conflicts of Interest

The authors declare no conflict of interest.

## Supporting information




**Supporting File**: advs73815‐sup‐0001‐SuppMat.docx.

**Supporting File**: advs73815‐sup‐0002‐Table S1.xlsx.

**Supporting File**: advs73815‐sup‐0003‐Table S2.xlsx.

**Supporting File**: advs73815‐sup‐0004‐Table S3.xlsx.

**Supporting File**: advs73815‐sup‐0005‐Table S4.xlsx.

**Supporting File**: advs73815‐sup‐0006‐Table S5.xlsx.

**Supporting File**: advs73815‐sup‐0007‐Table S6.xlsx.

**Supporting File**: advs73815‐sup‐0008‐Table S7.xlsx.

**Supporting File**: advs73815‐sup‐0009‐Table S8.xlsx.

## Data Availability

The raw sequence data reported in this study have been deposited at the National Genomics Data Center (NGDC), China National Center for Bioinformation (CNCB) (accession: PRJCA044567). All single‐cell transcriptome data analysis codes are available from the GitHub depository https://github.com/BingruZhao/Cross‐species‐single‐cell‐atlas.
