# Supplementary material for: Comparative Single‐Cell Transcriptomic Atlas Reveals the Genetic Regulation of Reproductive Traits
Source: Adv Sci (Weinh). 2026 Jan 21;13(17):e17633. doi: 10.1002/advs.202517633 (PMC13042767; doi:10.1002/advs.202517633)
Supplement: Supplementary file 1 — Supporting File: advs73815‐sup‐0001‐SuppMat.docx. [file ADVS-13-e17633-s006.docx]

**
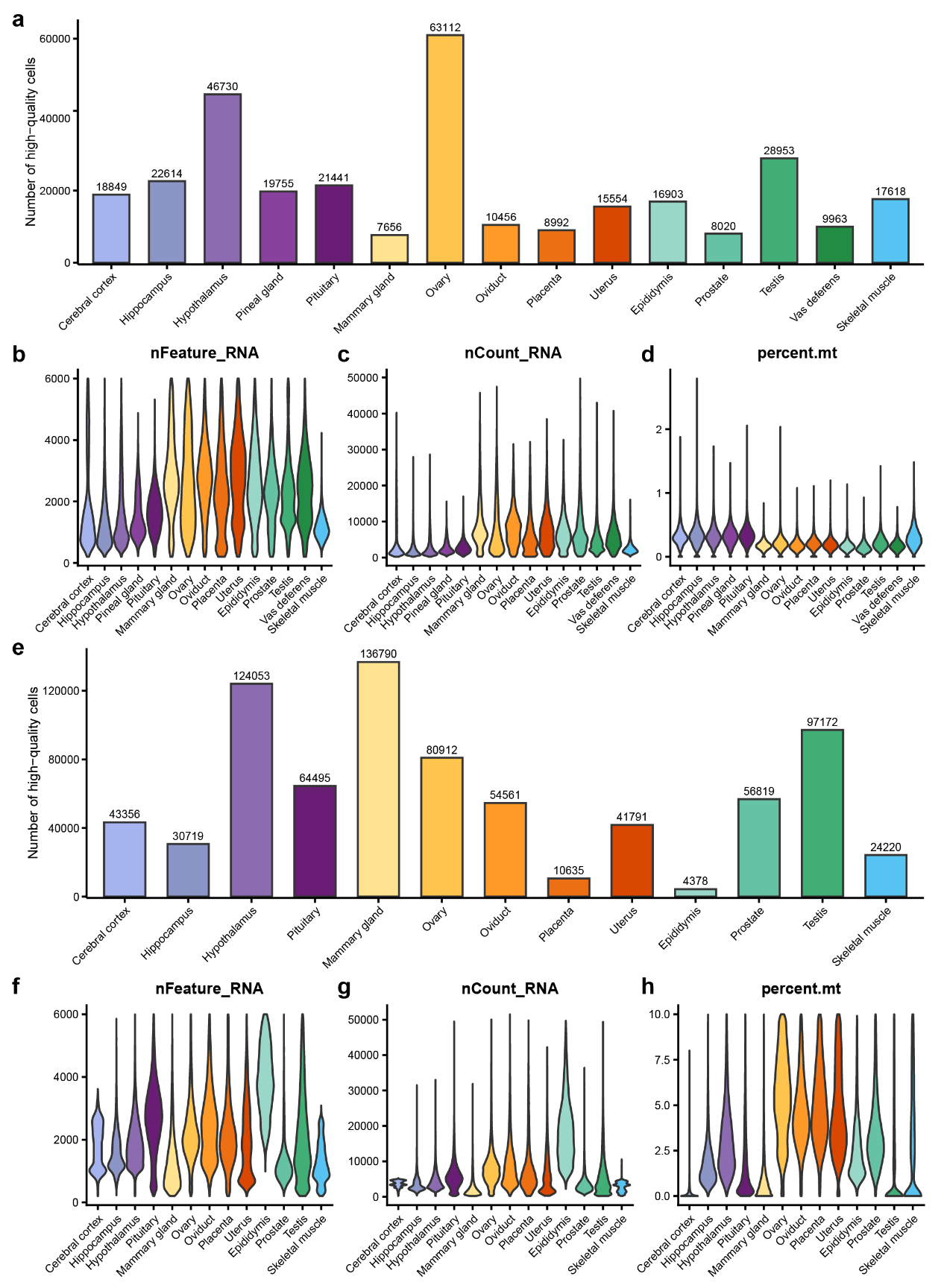
**

**Figure S1. Quality control of single-cell RNA-seq data from sheep and human tissues.** a–d, Sheep data; e–h, human data. The number of high-quality cells (a, e), genes detected per cell (**nFeature_RNA**; b, f), total UMI counts per cell (**nCount_RNA**; c, g) and mitochondrial transcript percentages (**percent.mt**; d, h) are shown for each tissue after quality control. UMI: Unique molecular identifier.


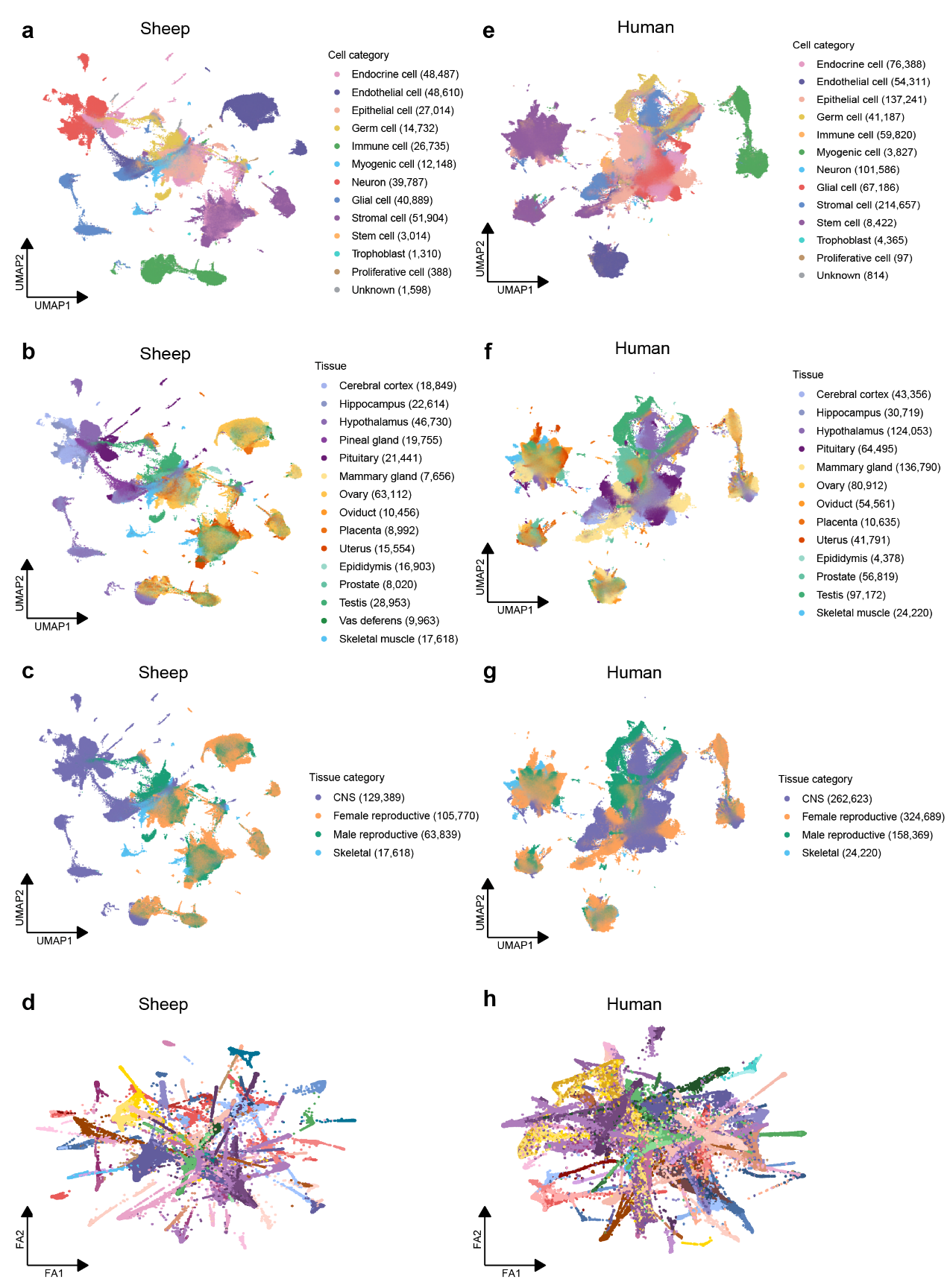


**Figure S2. UMAP visualization and lineage trajectory analysis.** a–d, Sheep; e–h, humans. UMAP plots showing all identified cell clusters colored by cell categories (a, e), tissues (b, f), and tissue categories (c, g). (d, h) Branching gene expression trajectories visualized by PAGA, colored by annotated cell types in sheep (d) and humans (h).


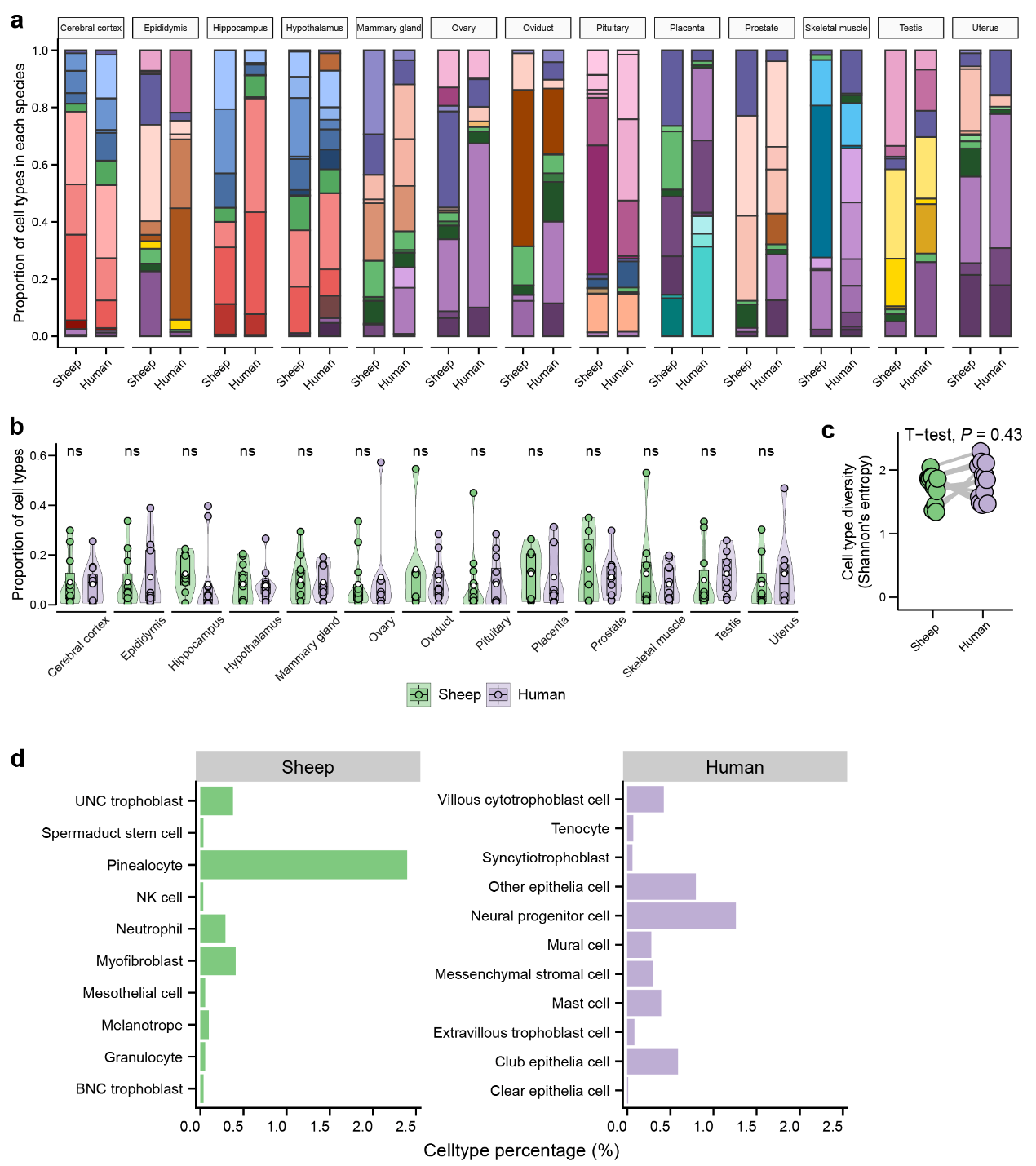


**Figure S3. Comparison of cell-type composition and diversity between sheep and human tissues.** (a) Stacked bar plots showing the relative proportions of cell types within each tissue for sheep and humans. (b) Violin plots comparing the proportions of shared cell types within each tissue between species (two-sided Student’s t-test, all comparisons were non-significant (ns), *P* > 0.05). (c) Cell-type diversity (measured by Shannon entropy) of each tissue in each species (two-sided t-test, *P* = 0.43). Solid lines connect corresponding tissues between species. (d) Proportions of species-specific cell types in sheep and human datasets.


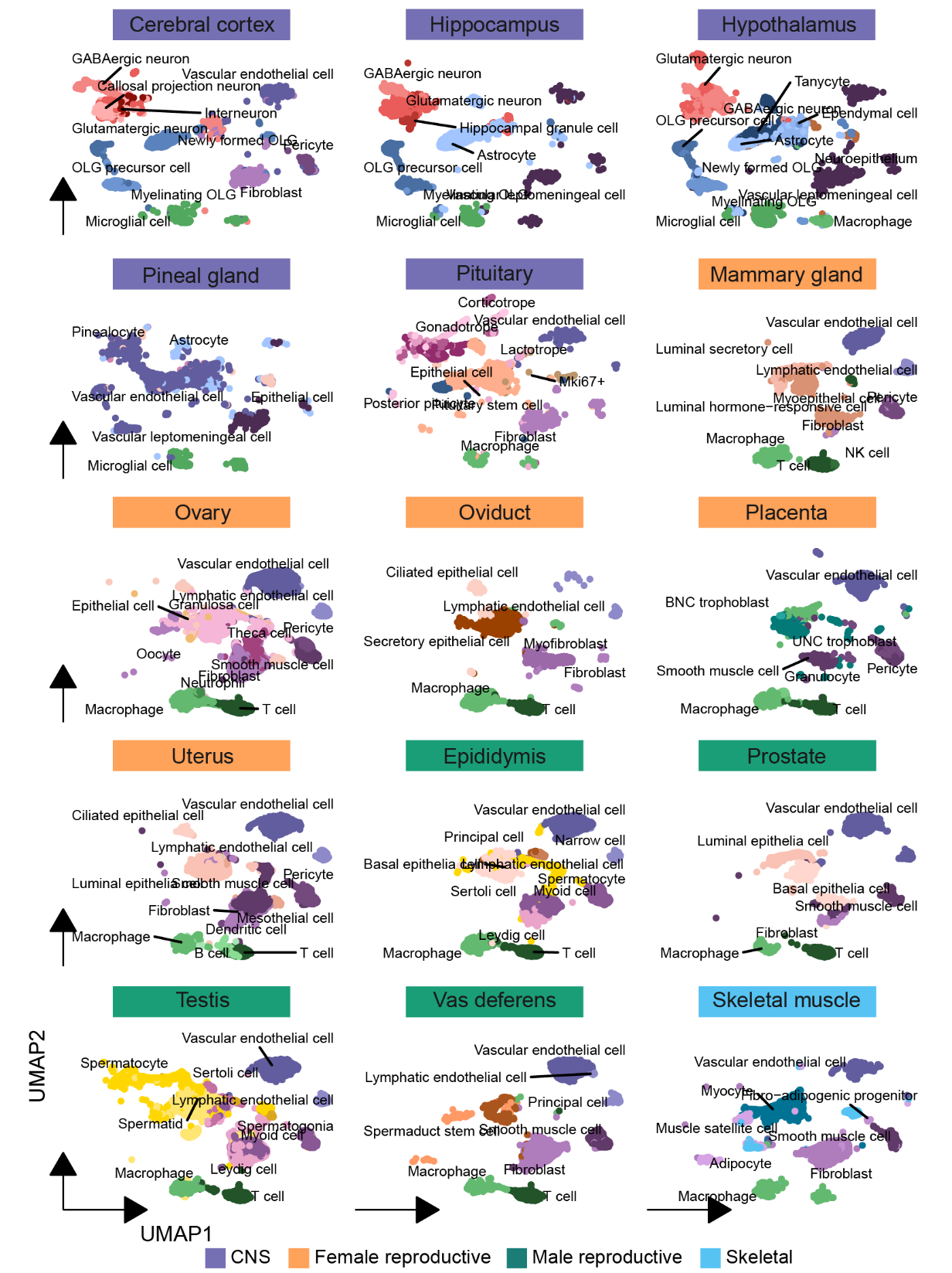


**Figure S4. UMAP visualization of cell clusters from 15 sheep tissues.** Tissues are classified into four categories, including central nervous system (CNS), female reproductive, male reproductive, and skeletal, and the top labels of UMAP plots are colored according to categories.


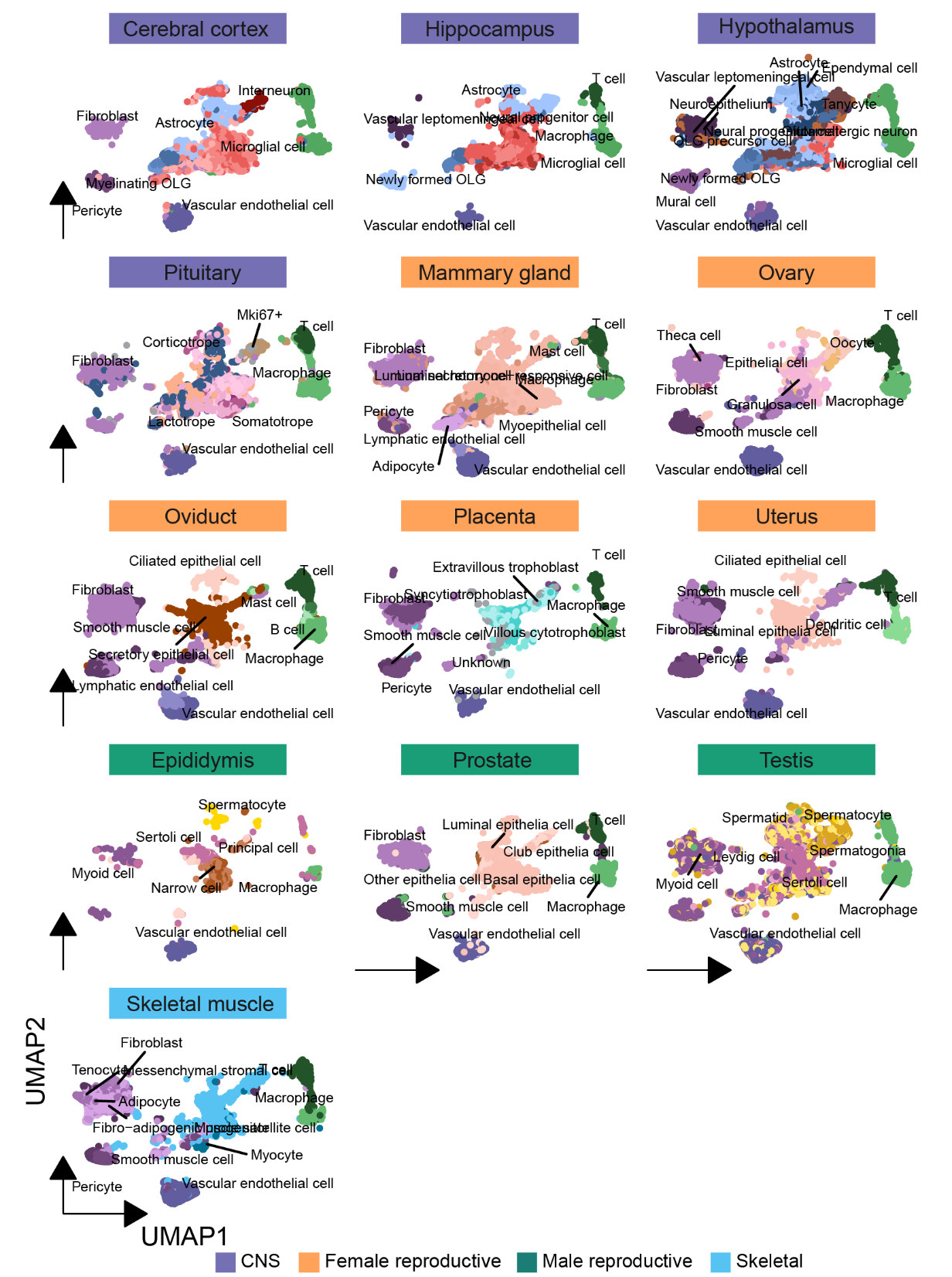


**Figure S5. UMAP visualization of cell clusters from 13 human tissues.** Tissues classified into four categories, including central nervous system (CNS), female reproductive, male reproductive, and skeletal, and the top labels of UMAP plots are colored according to categories.

**
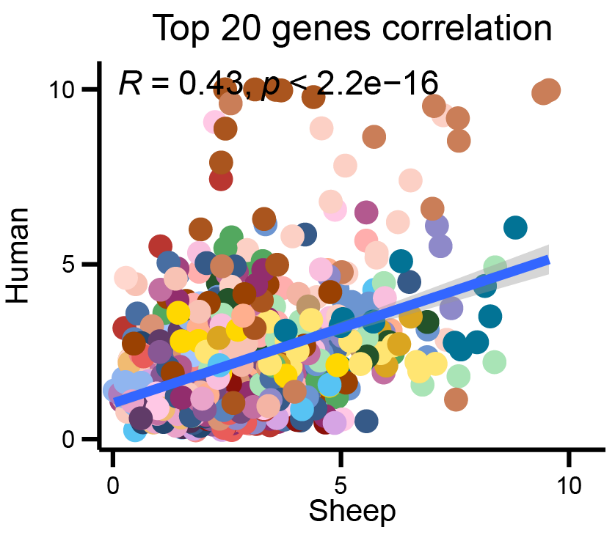
**

**Figure S6. Scatter plot showing the correlation of the top 20 marker genes identified by Seurat used for cell-type annotation between sheep (x-axis) and humans (y-axis).**
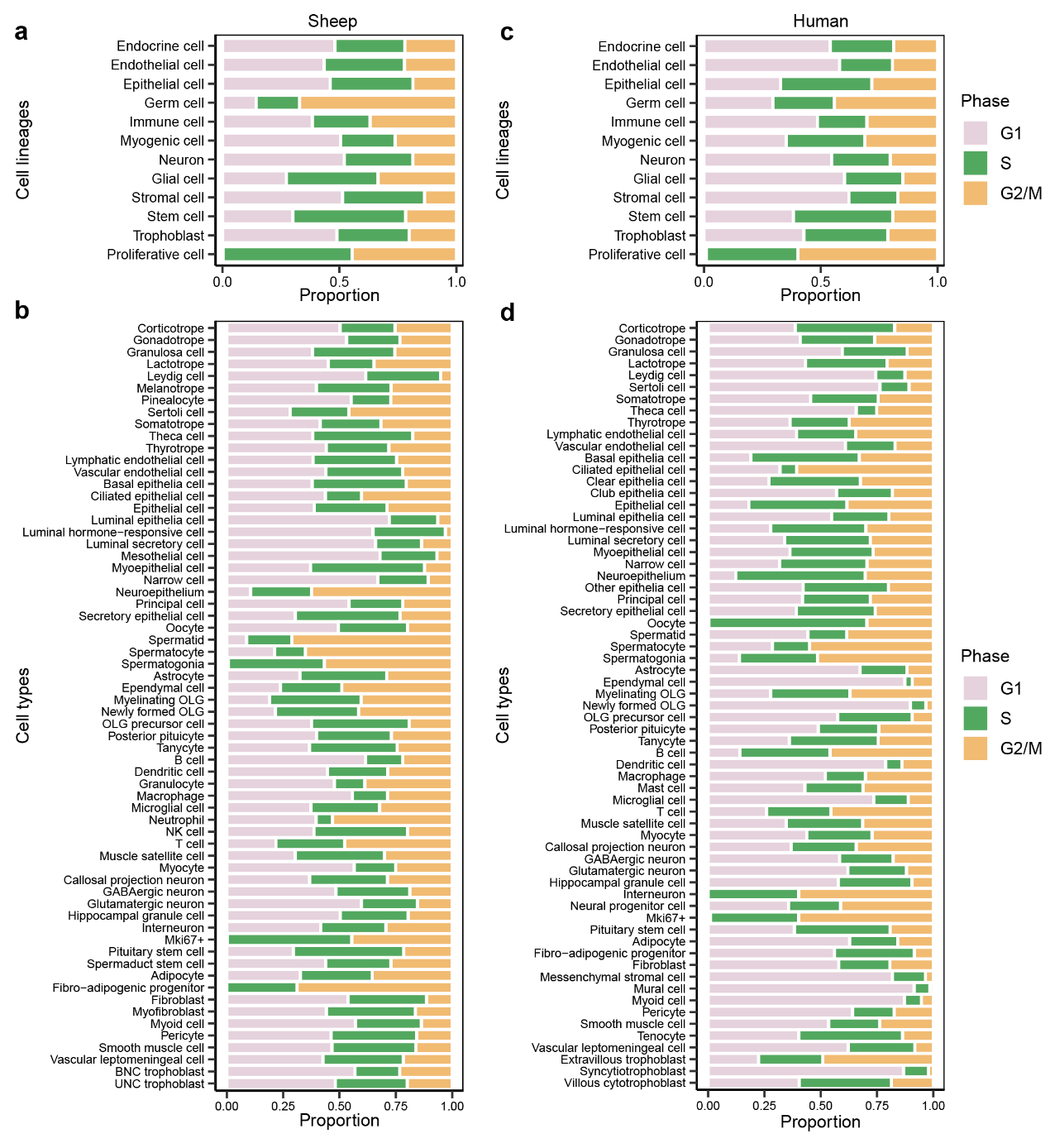


**Figure S7. Cell-cycle analysis across cell lineages in sheep and humans.** a and b for sheep data; c and d for human data. Bar plots showing the cell-cycle phase (G1, S, and G2M) distributions across broad cell lineages (a, c) and detailed cell types (b, d). G1 (Gap 1 Phase): cell growth and preparation for DNA synthesis; S (Synthesis Phase): DNA replication; G2M (Gap 2 to Mitosis Phase): preparation and initiation of mitosis.

**
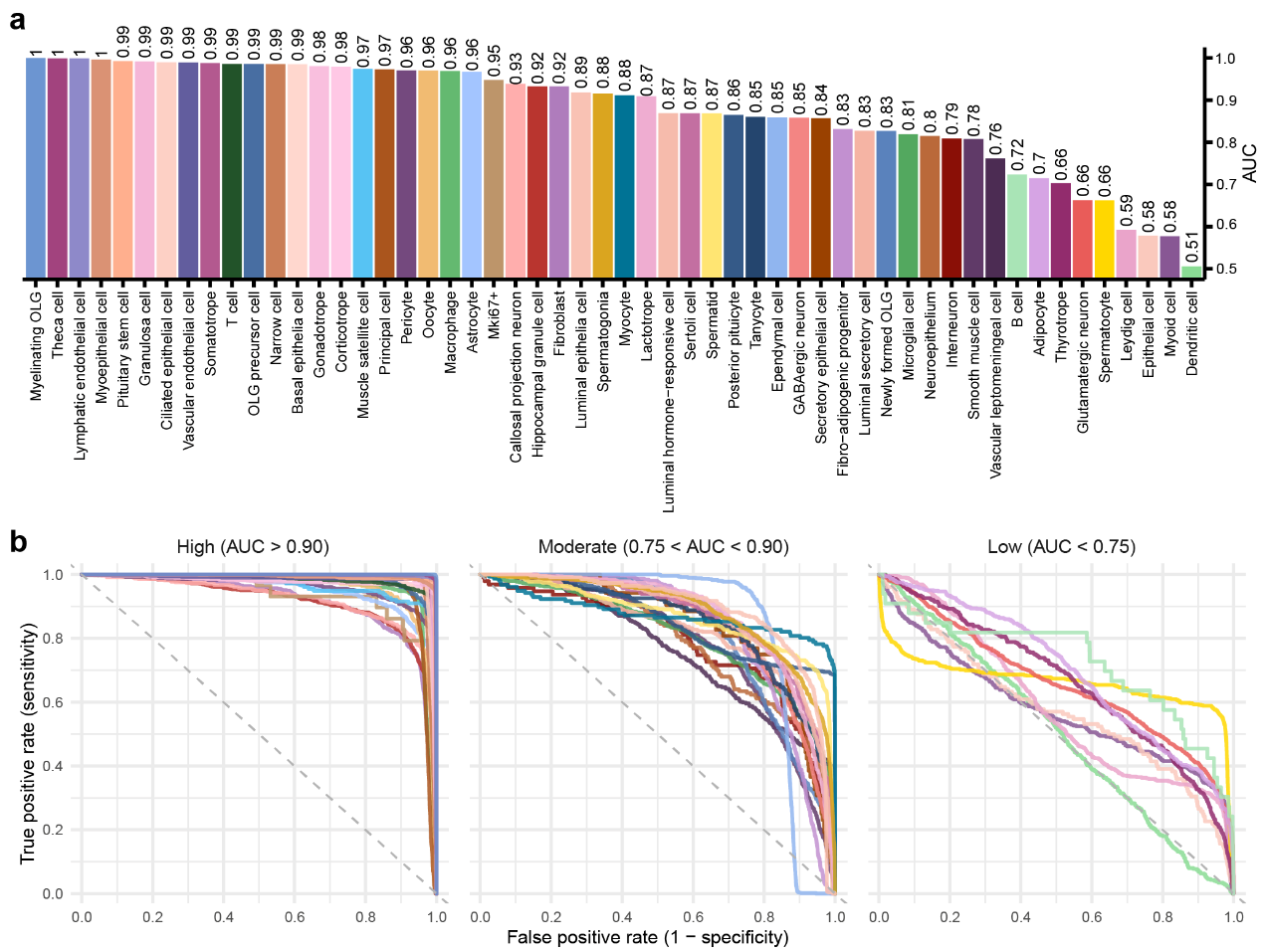
**

**Figure S8. Cross-species cell-type prediction accuracy.** **(**a) Cell types ranked by classification performance (AUC) from sheep to human predictions. (b) ROC curves stratified by classifier performance: high (AUC ≥ 0.90), moderate (0.75 ≤ AUC < 0.90), and low (AUC < 0.75).

**
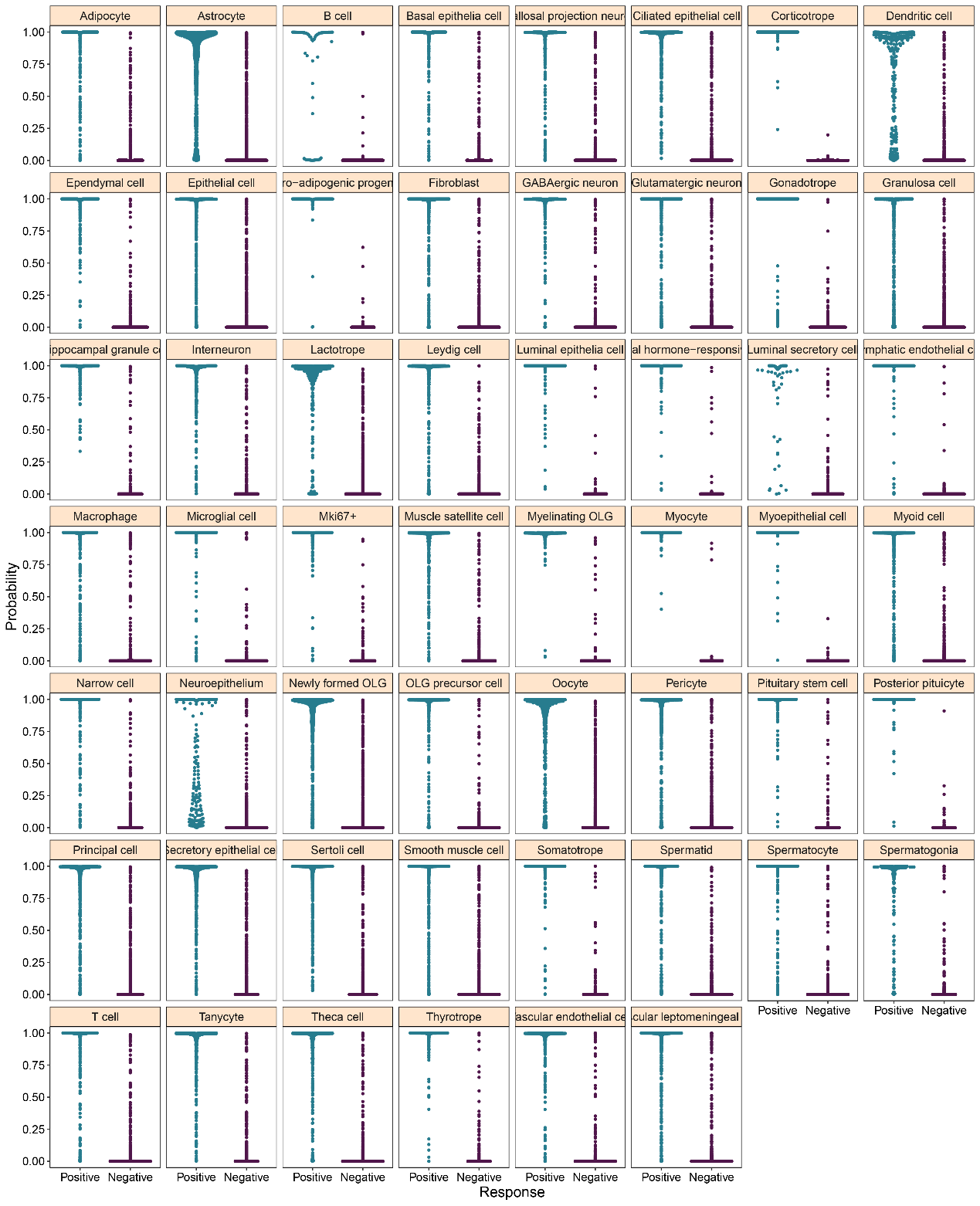
**

**Figure S9. Violin plots showing distributions of predicted probabilities for positive and negative cells across human cell types.** Most predictions exhibited strong separation, indicating high classifier confidence.


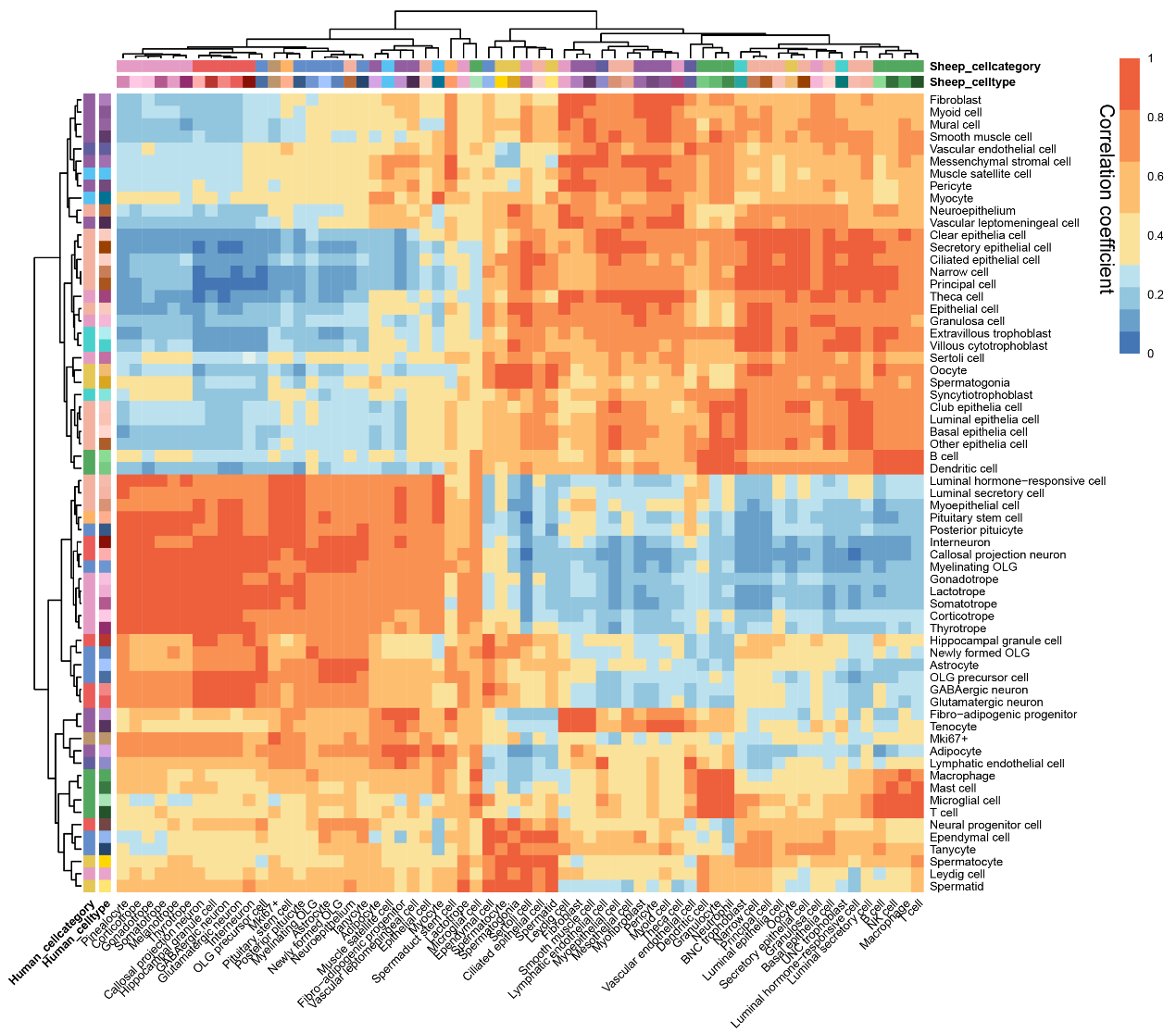


**Figure S10. Heatmap showing the correlation of orthologous gene expression between sheep and human cell types based on AUROC scores calculated from Spearman correlations of pseudo-cells (randomly selected 100 cells per cell type per species).**

**
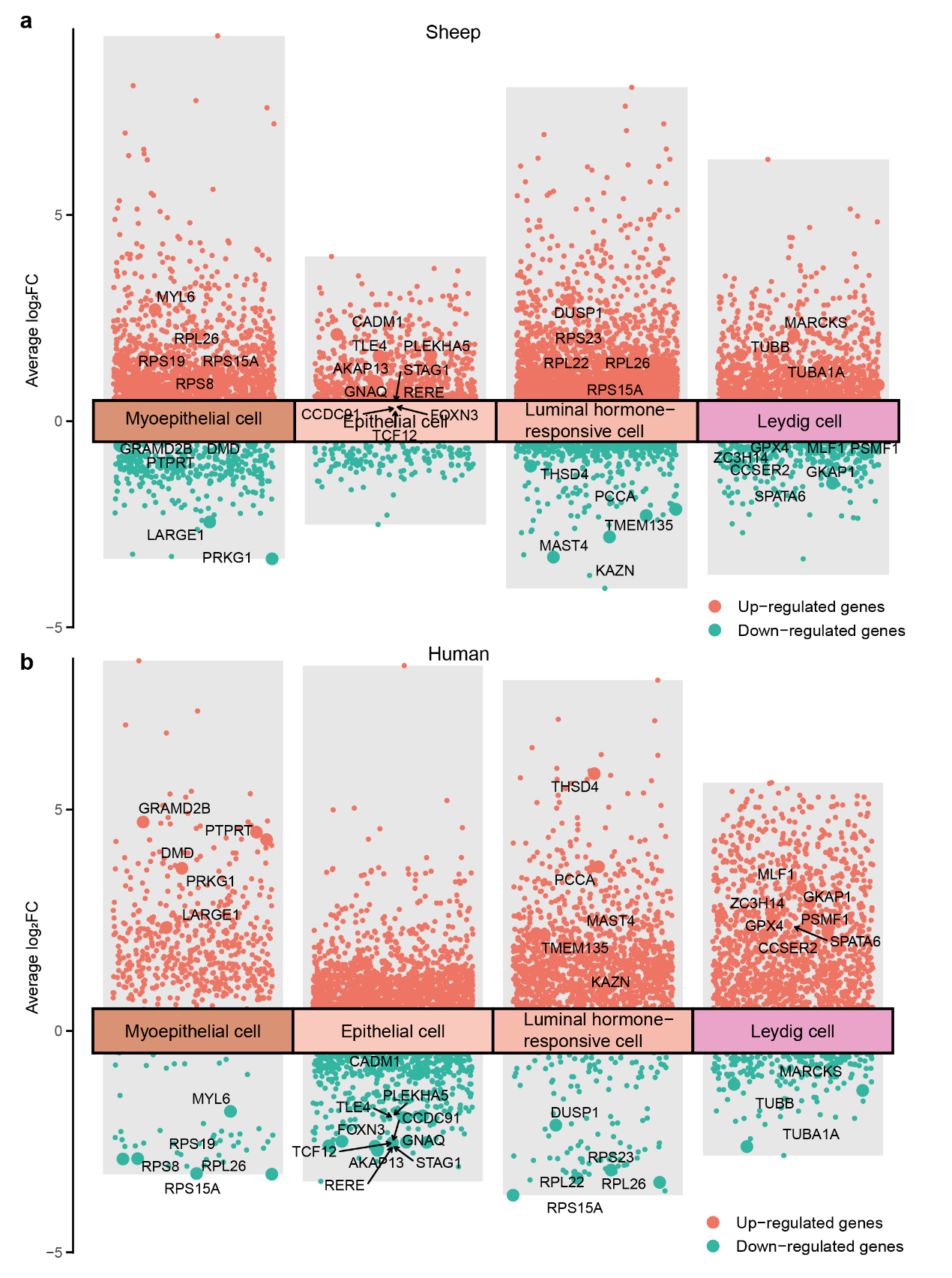
**

**Figure S11. Scatter plot showing differentially expressed genes (DEGs) identified in myoepithelial cells, epithelial cells, luminal hormone-responsive cells, and leydig cells in sheep (a) and humans (b).**


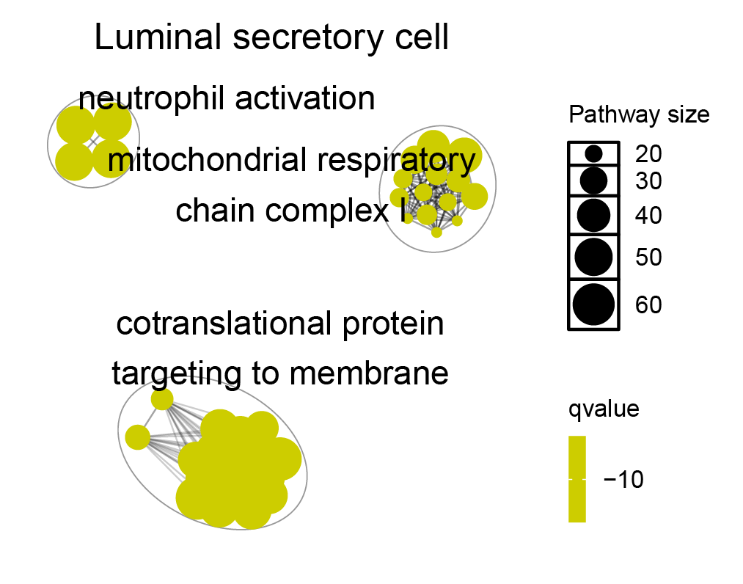


**Figure S12. Gene ontology (GO) enrichment analysis of upregulated genes in sheep luminal secretory cells compared to those of humans.**


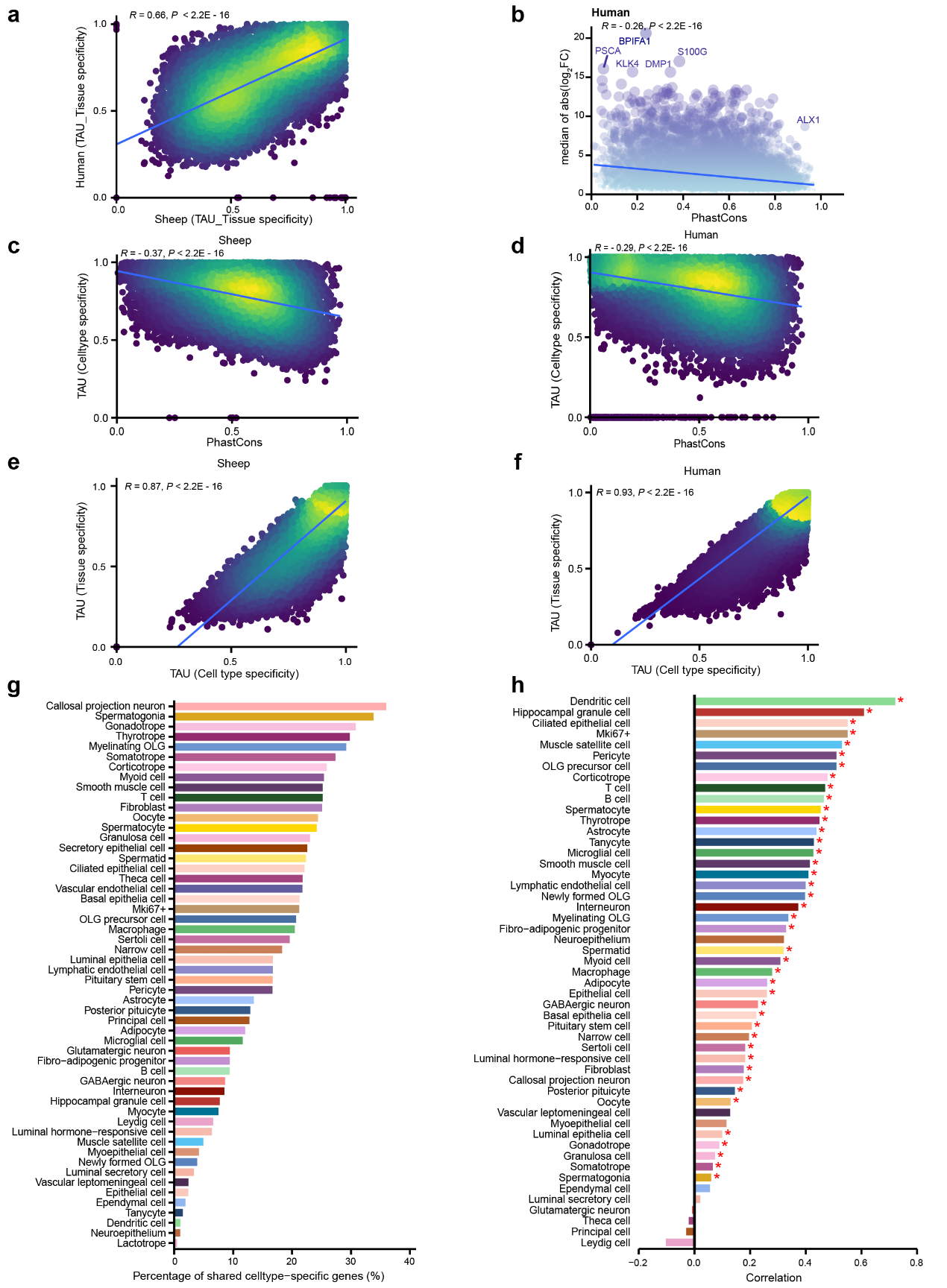


**Figure S13. Cross-species conservation of cell-type-specific gene expression. (a)** Correlation of tissue-specific tau values between sheep and humans. (b) Correlation between phastCons scores (sequence conservation) and median absolute log₂ fold change (log₂FC) in human cells. (c, d) Correlations between cell type-specific tau values and phastCons scores in sheep (c) and humans (d). (e, f) Correlation between cell type-specific and tissue-specific tau values in sheep (e) and humans (f). (g) Percentage of shared cell type-specific genes between sheep and humans across conserved cell types. (h) Spearman’s correlation of cell type-specific genes (measured by −log_10_*P* from cell type specificity expression analysis) between sheep and humans. “*” represents the correlation coefficient is significant (FDR < 0.05).

**
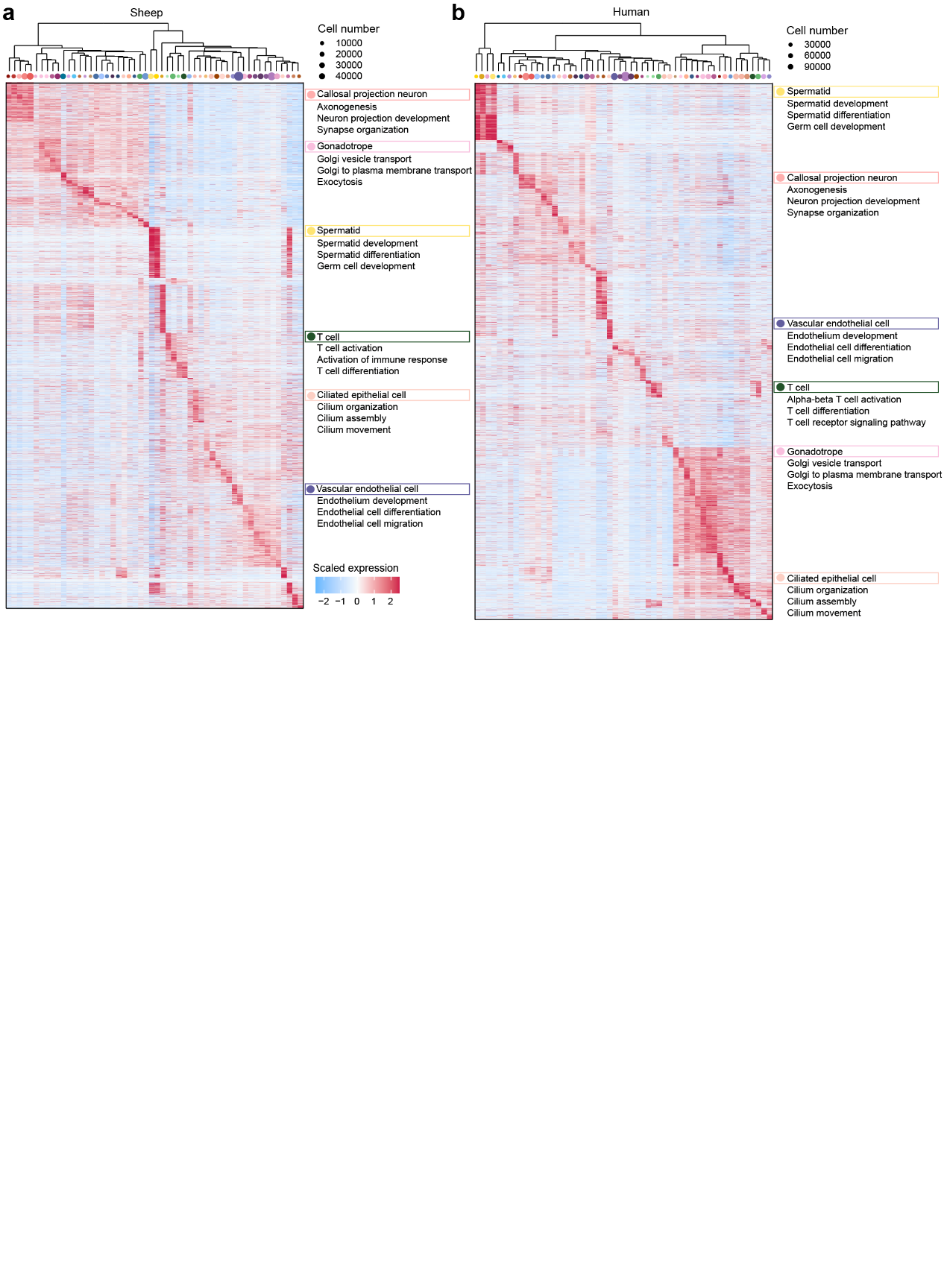
**

**Figure S14. Conserved expression patterns of cell type-specific genes between sheep and humans. Heatmaps showing the scaled expression (z-score) of cell type–specific genes in sheep (a) and humans (b). Each row represents a gene, and each column represents a cell type. The dot size above each column reflects the cell number within each cell type. Representative functional annotations are provided for selected cell type–specific genes.**

**
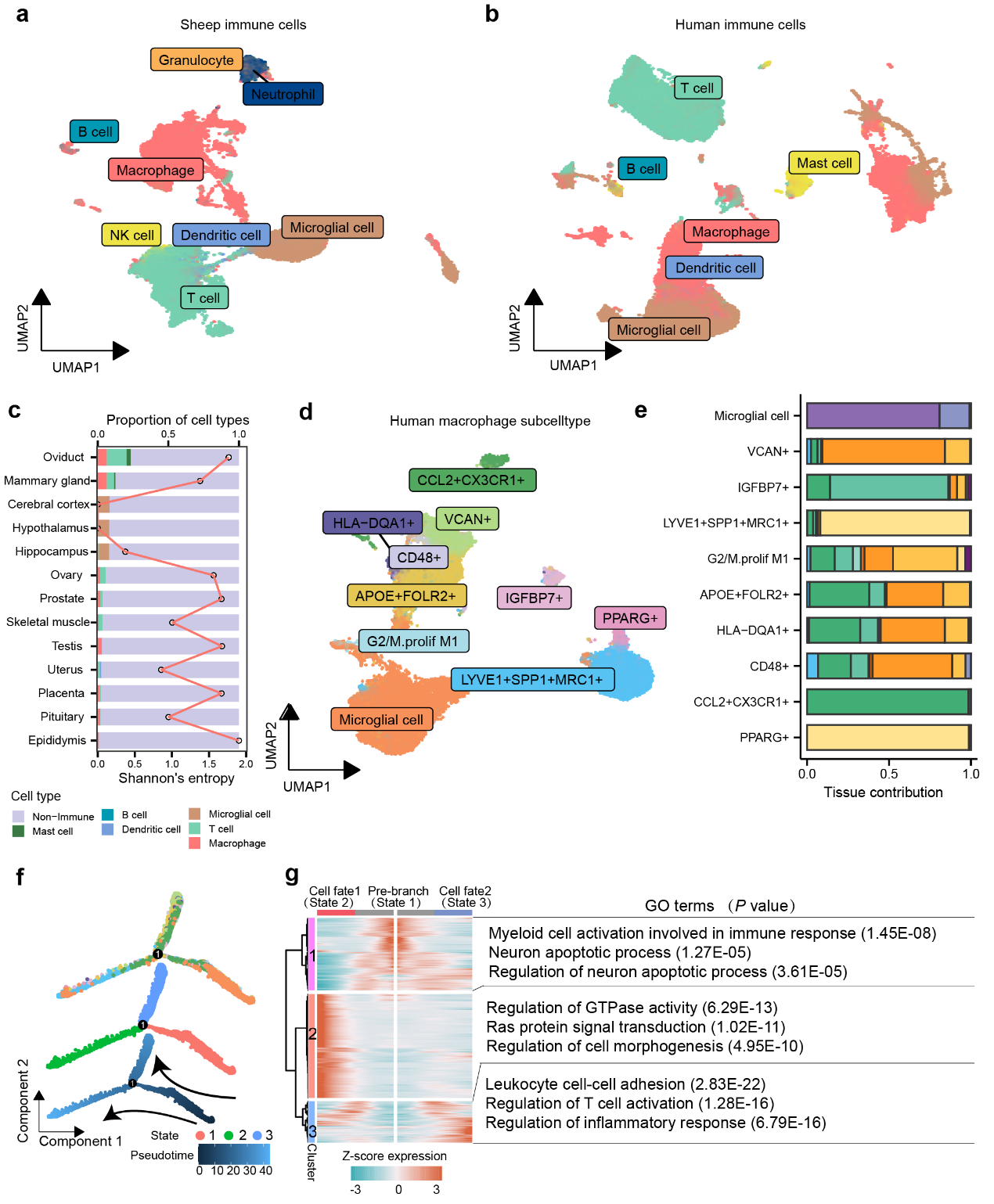
**

**Figure S15. Heterogeneity of immune cells across tissues in sheep and humans.** (a, b) UMAP plots of immune cells in sheep (a) and humans (b), colored by cell type. (c) Proportions of immune and non-immune cell types across human tissues. The red line represents the Shannon entropy values for immune cells in each tissue, reflecting the degree of immune cell diversity within each tissue. (d) UMAP visualization of macrophage subtypes in humans. (e) Tissue contributions to each human macrophage subtype. (f) Pseudotime trajectories of human macrophages, colored by modeled pseudotime (bottom), inferred states (middle), and subtypes (top). (**g**) **Pseudo-heatmap of differentially expressed genes (DEGs) during the macrophage cell fate commitment (left), and** top enriched Gene Ontology (GO) terms (P < 0.05) **for each gene set (right) in** humans**.**


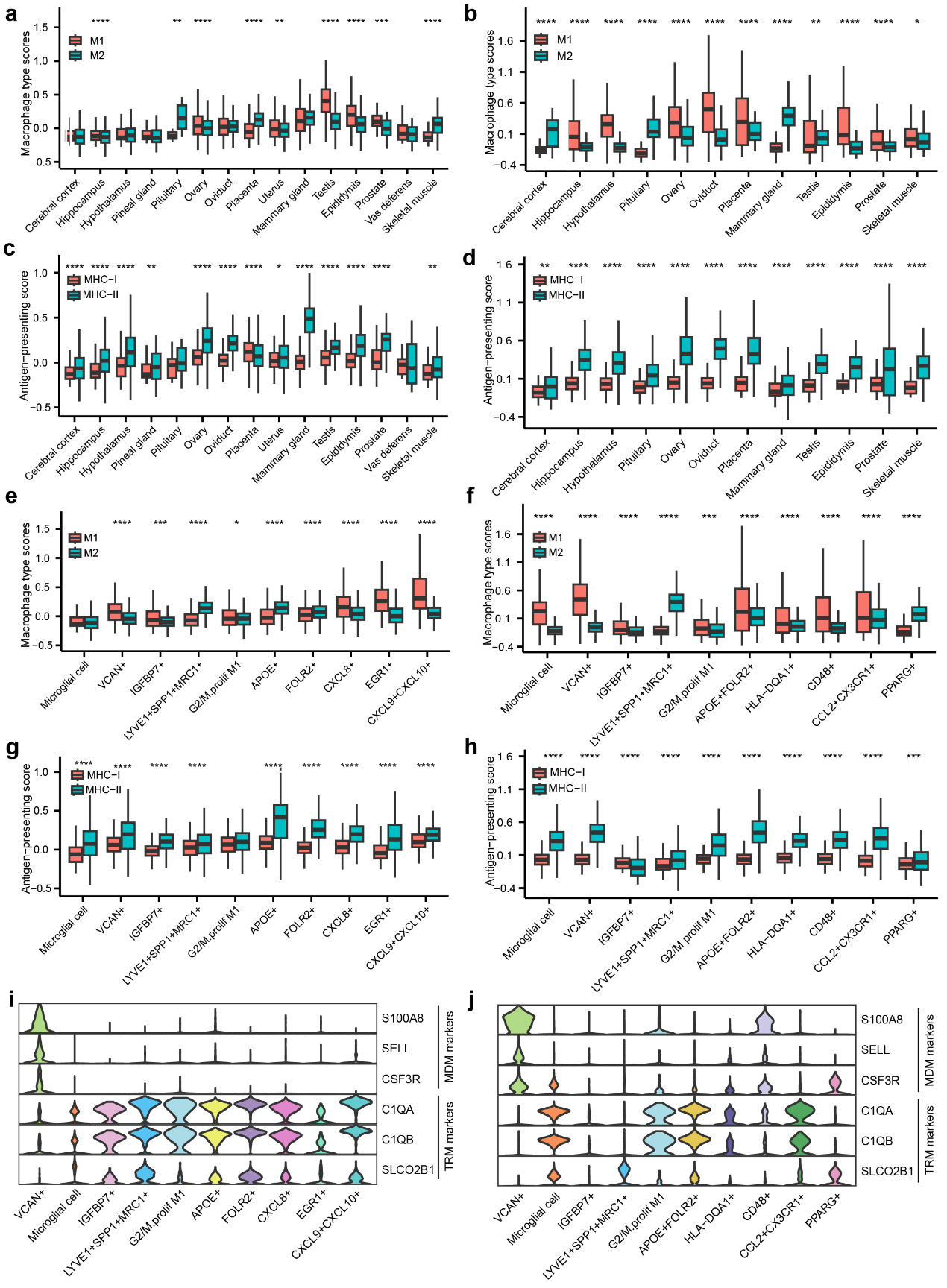


**Figure S16. Cross-species comparison of macrophage polarization, antigen presentation, subtype specialization, and developmental origin. (a, b)** M1 and M2 macrophage polarization scores across tissues in sheep (a) and humans (b). **(c, d) The antigen-presenting scores for intracellular (MHC I) and extracellular (MHC II) antigens across tissues in sheep (c) and humans (d). (e, f)** M1 and M2 polarization scores across macrophage subtypes in sheep (**e**) and humans (**f**), including five macrophage subtypes (Microglial cell, VCAN⁺, IGFBP7⁺, LYVE1⁺SPP1⁺, MRC1⁺, and G2/M.prolif M1) and species-specific cell subtypes. **(g, h)** MHC-I and MHC-II antigen-presenting scores across macrophage subtypes in sheep (g) and humans (h). **(i, j) The violin plot showing the expression levels of** canonical monocyte-derived macrophage (MDM) **marker genes (*S100A8*, *SELL*, *CSF3R*) and** tissue-resident macrophage (TRM) **marker genes (*C1QA*, *C1QB*, *SLC02B1*) across macrophage subtypes in sheep (i) and humans (j).** Statistical significance was calculated using the two-sided unpaired Student’s t-test, with *, **, *** and **** indicating *P*-value < 0.05, 0.01, 0.001, and 0.0001, respectively.


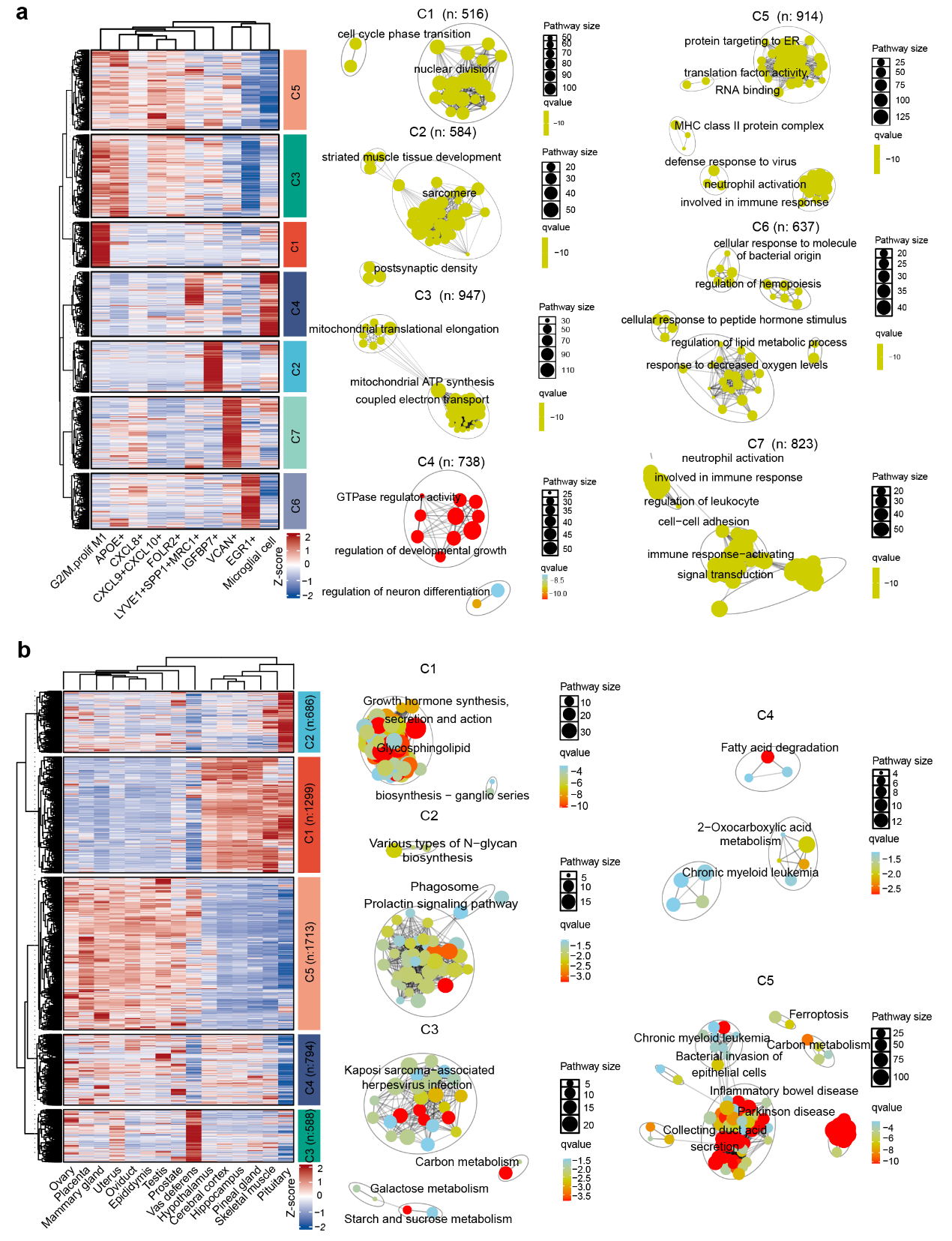


**Figure S17. Clustering analysis of subtype- and tissue-specific gene expression in macrophages. (a)** Heatmap showing clustering of macrophage subtype–specific genes. (**b**) Heatmap showing the clustering of tissue-specific genes across macrophage populations. For each heatmap, representative gene clusters were analyzed for KEGG enrichment. Enriched pathways are visualized as functional networks, with node size representing gene set size and color indicating statistical significance (FDR < 0.05).

**
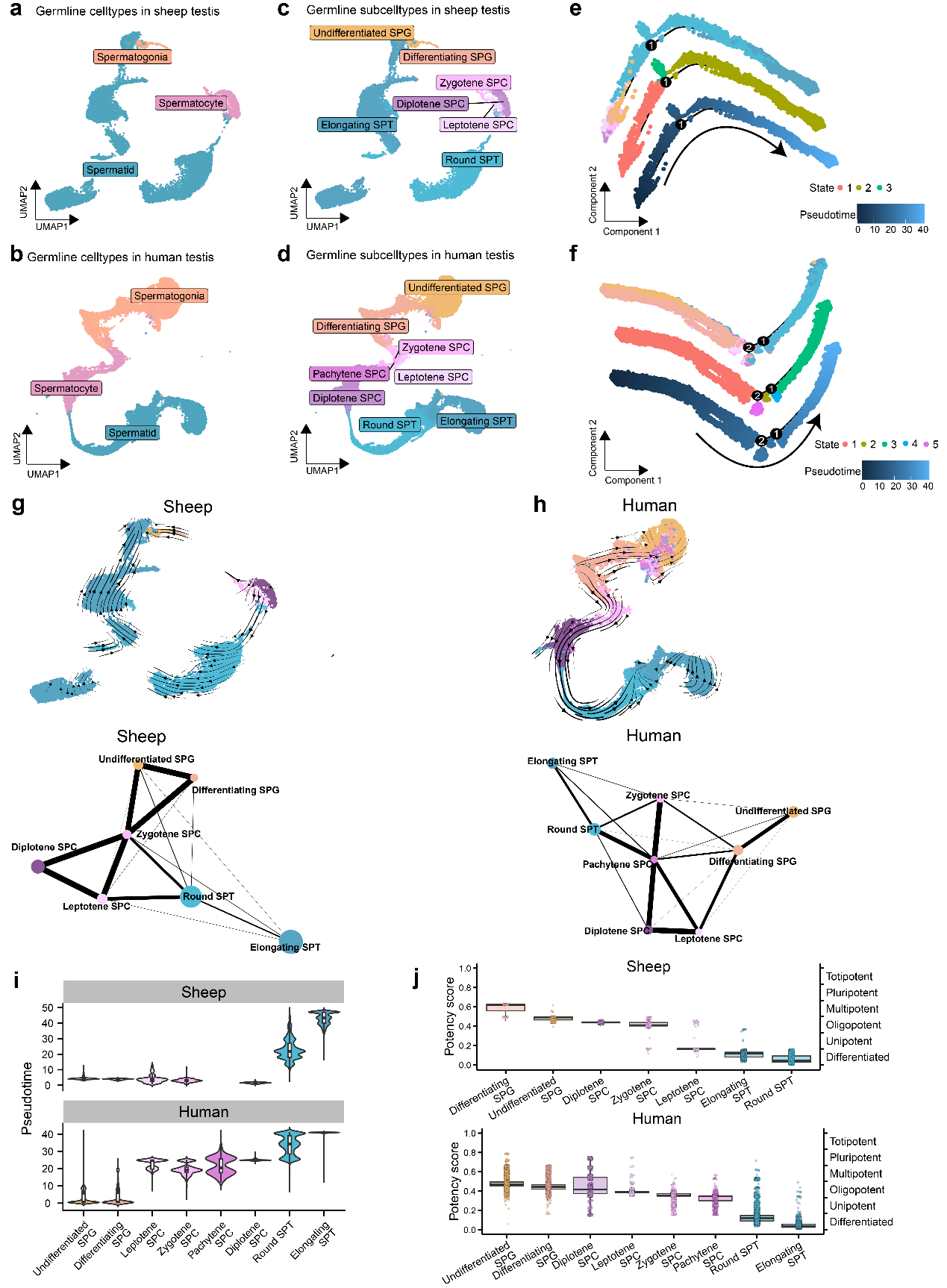
**

**Figure S18. Cross-species comparison of spermatogenic cell differentiation. (a–d)** UMAP visualization of major germline cell types (a, b) and annotated subtypes (c, d) in sheep (a, c) and humans (b, d) in testis. Cells were categorized into spermatogonia (SPG), spermatocytes (SPCs), and spermatids (SPTs), with finer resolution into developmental subtypes. **(e, f)** Pseudotime trajectories showing a continuous differentiation from undifferentiated SPG to elongating SPTs in sheep (e) and humans (f). **(g, h)** RNA velocity (top) and PAGA connectivity graphs (bottom) showing directional progression along the germline trajectory in sheep (g) and humans (h). **(i)** Violin plots of pseudotime values across germline subtypes. **(j)** Boxplots

of CytoTRACE scores, showing stepwise loss of developmental potential from undifferentiated SPG to mature SPTs.


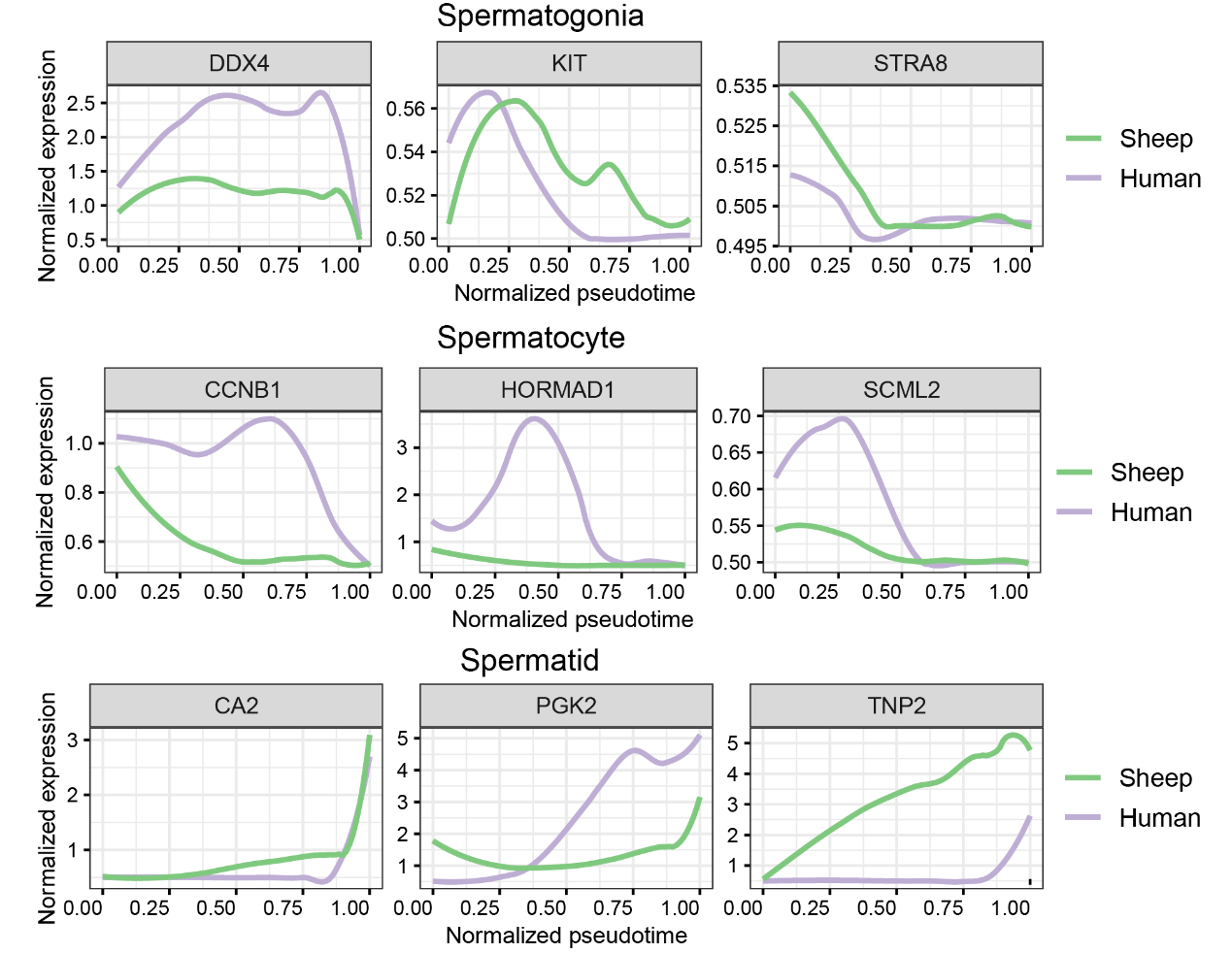


**Figure S19 Conserved expression dynamics of spermatogenic cell markers along pseudotime.** Normalized expression of representative marker genes for spermatogonia (DDX4, KIT, STRA8), spermatocytes (CCNB1, HORMAD1, SCML2), and spermatids (CA2, PGK2, TNP2) along pseudotime in sheep (green) and humans (purple), highlighting conserved transcriptional programs in spermatogenesis.

**
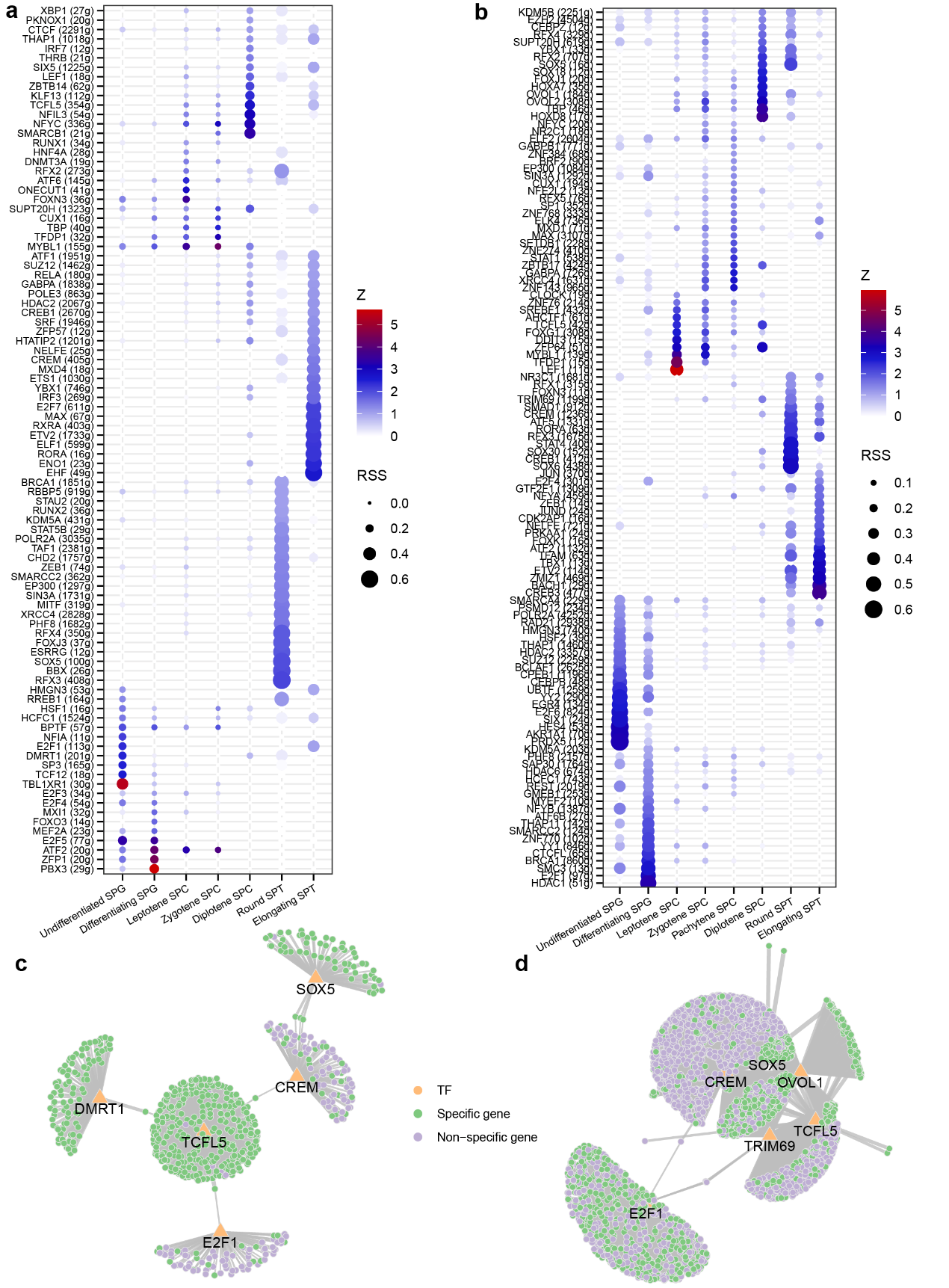
**

**Figure S20. Identification of subtype-specific transcription factors (TFs) in sheep and human spermatogenic cells.** (a, b) Bubble plots showing representative TFs (RSS > 0.1) enriched in specific germline subtypes from undifferentiated spermatogonia (SPG) to elongating spermatids (SPTs) in sheep (a) and humans (b). Dot size represents the regulon specificity score (RSS), and color indicates expression Z-score. (c, d) TF-target gene networks for sheep (c) and human (d) germline cells, highlighting both conserved TFs across species (e.g., **E2F1**, **SOX5**, **CREM**, **TCFL5**) and species-specific TFs (e.g., **DMRT1** in sheep; **TRIM69**, **OVOL1** in humans).


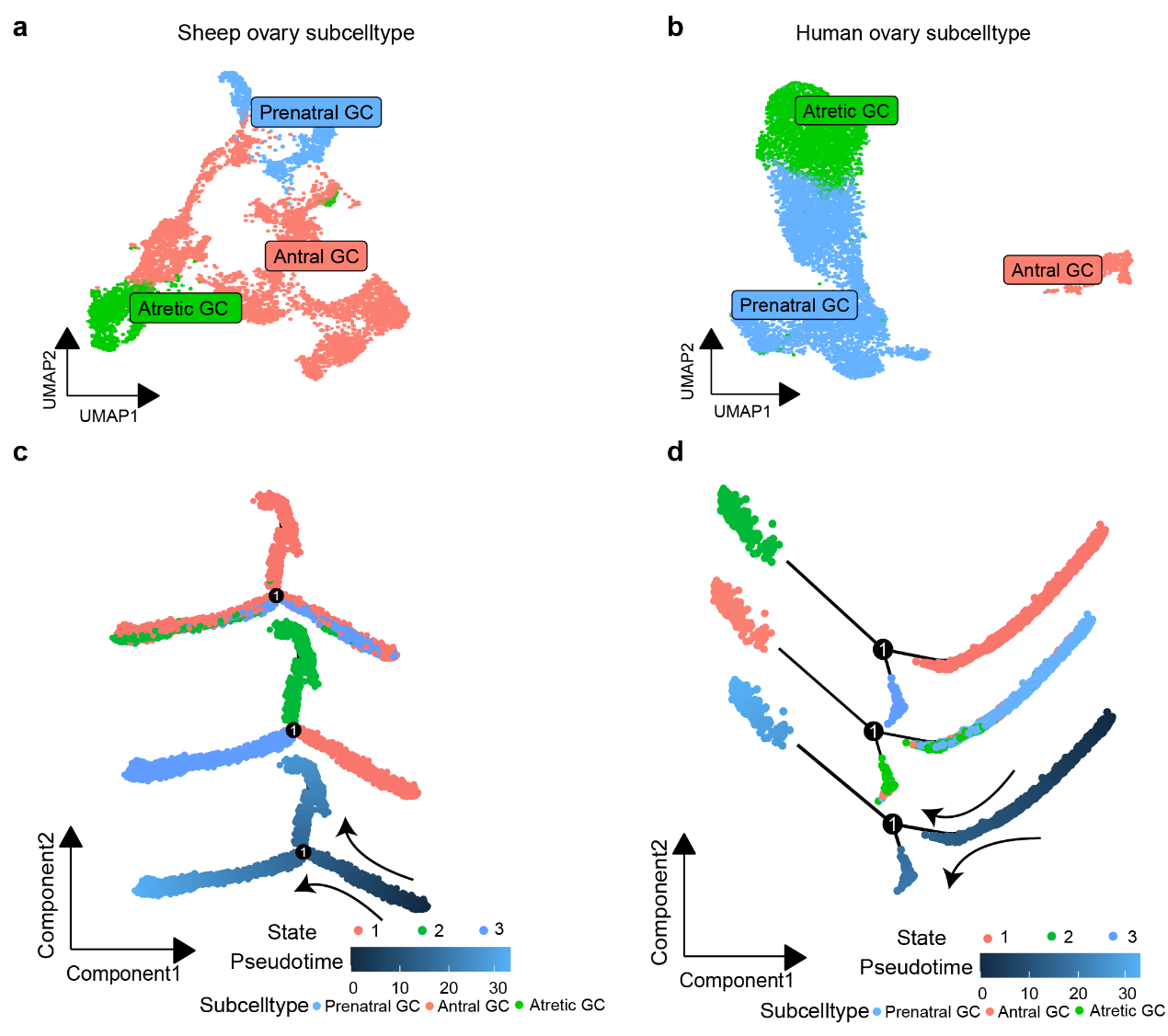


**Figure S21. Cross-species analysis of granulosa cell (GC) subtypes and differentiation trajectories in the ovary.** (a, b) UMAP visualization of GC subtypes in sheep (a) and humans (b), classified into three subtypes: preantral, antral, and atretic GCs based on marker gene expressions. Preantral GCs showed high expressions of marker genes such as *IGFBP5*, *GATM*, and *COL18A1*; antral GCs were marked by *INHBB*, *FST* and *GJA1*; and atretic GCs by *ITIH5* and *GHR*. (c, d) Pseudotime trajectory analysis of GC subtypes in sheep (c) and humans (d). Pseudotime trajectories of GCs in sheep (c) and humans (d), colored by modeled pseudotime (bottom), predicted cell states (middle), and subtypes (top).


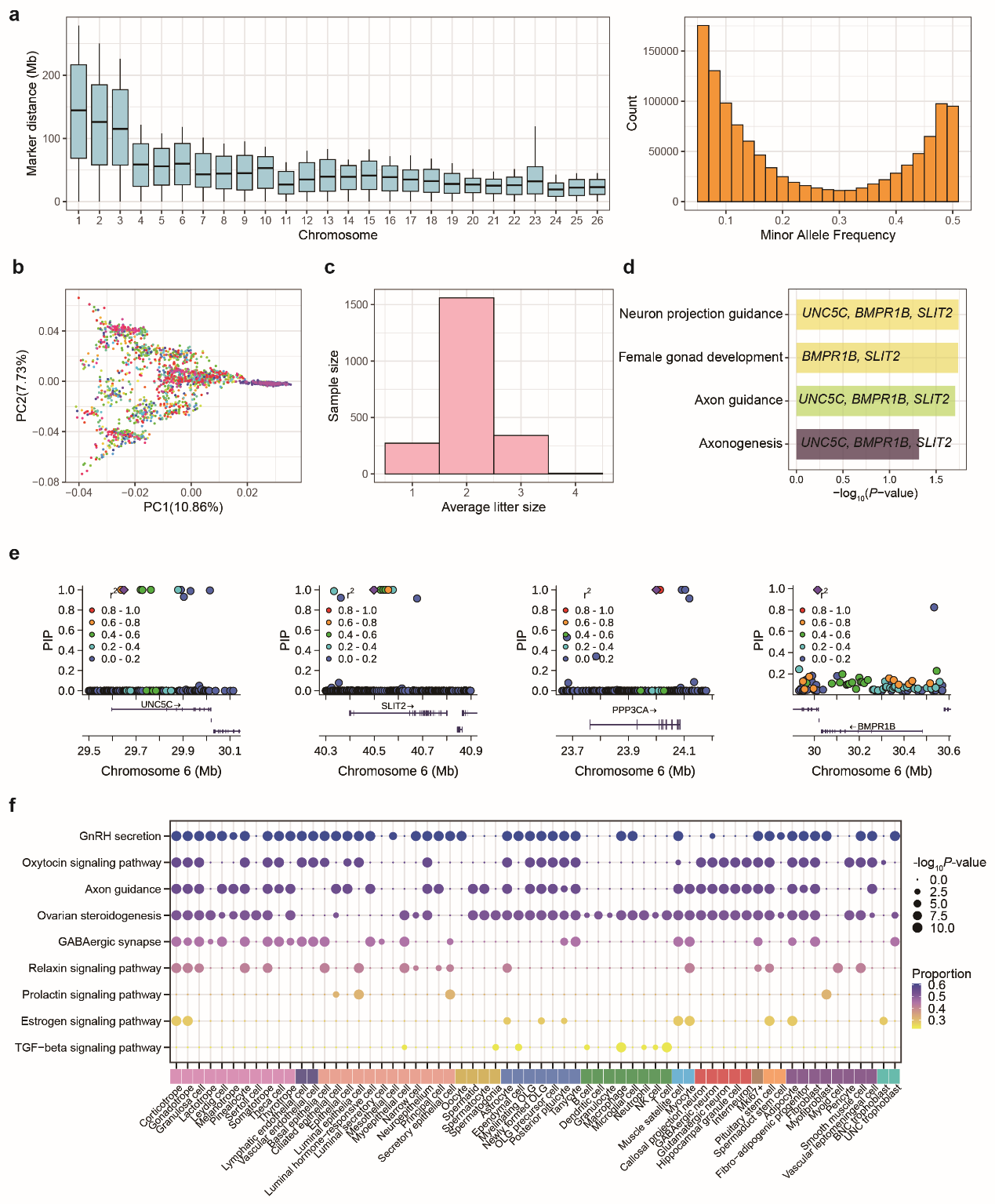


**Figure S22 Genomic features, association signals, and cell-type expression profiles from sheep fertility GWAS.** (a) Distribution of genetic markers and allele frequencies in the imputed dataset. Left: Inter-marker distances (Mb) across the 26 autosomes. Right: Minor allele frequency (MAF) spectrum for SNPs retained after imputation and quality control (n = 1,152,006). (b) Principal component analysis (PCA) of all sequenced individuals (n = 2,180) based on genome-wide SNPs. (c) Histogram of lifetime average litter size recorded across the study cohort. (d) GO enrichment of candidate genes from SNP- and gene-based GWAS analysis. (e) Fine-mapping of four representative GWAS loci (*UNC5C*, *SLIT2*, *PPP3CA*, *BMPR1B*) on chromosome 6. For each region, posterior inclusion probabilities (PIP) and local linkage disequilibrium (r²) are shown for SNPs in the credible set. (f) Dot plots of trait-relevant pathways associated with lifetime average litter size in sheep identified by scPagwas. Dot size represents the significant level (-log_10_*P*-value) for each pathway, and color intensity indicates the proportion of cells within each type influenced by these pathways (pathway-level coefficient β > 0).


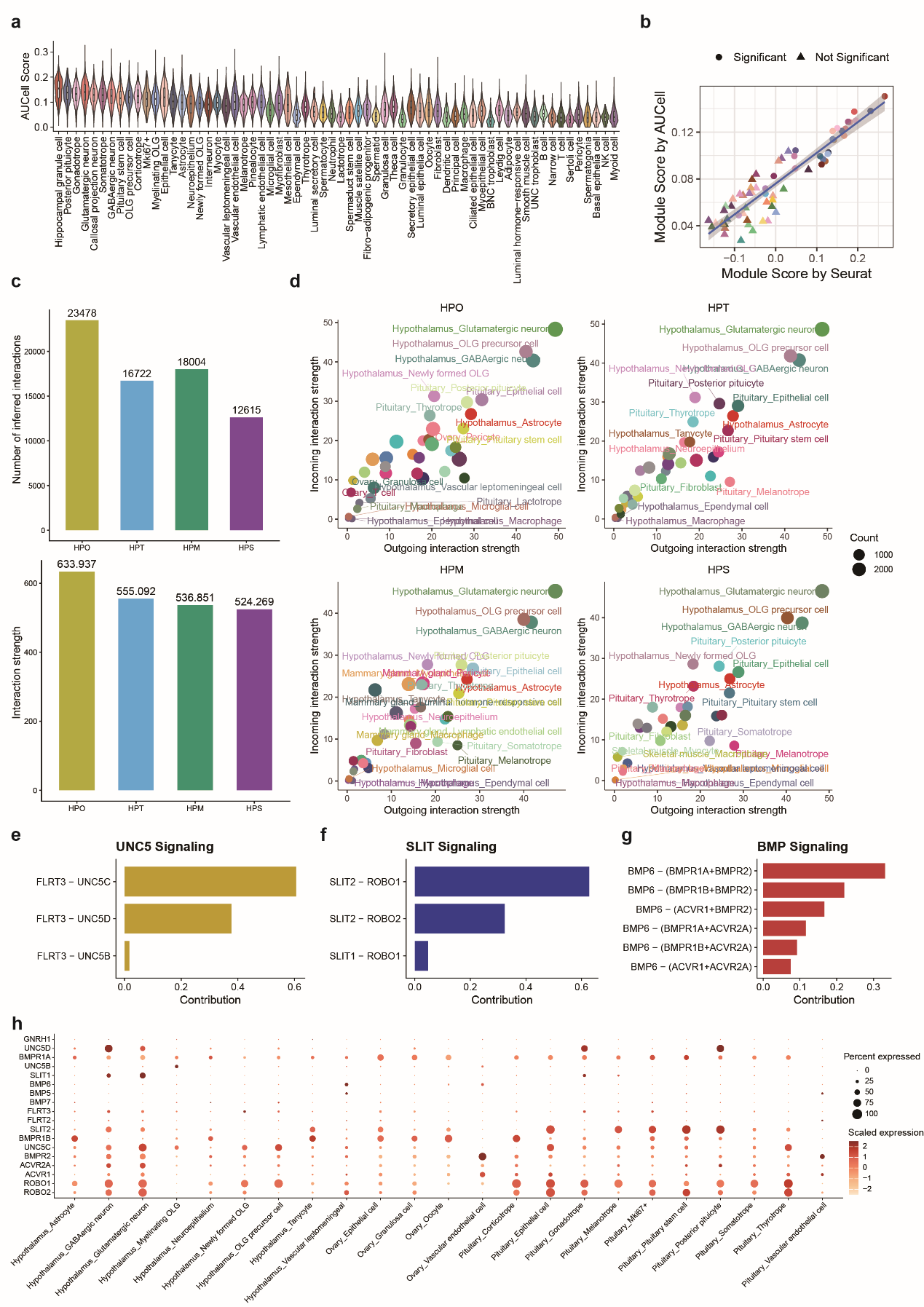


**Figure S23 Cell–cell communication dynamics and ligand–receptor contributions of UNC5, SLIT, and BMP pathways along the HPO axis.** (a) Violin plots showing AUCell-derived module activity scores for GWAS candidate genes across 65 sheep cell types. (b) Correlation between module scores calculated using AUCell and AddModuleScore. Each dot represents a cell type. (c) Number of inferred interactions (top) and interaction strength (bottom) for four major tissue axes, including hypothalamus–pituitary–ovary (HPO), hypothalamus–pituitary–testis (HPT), hypothalamus–pituitary–mammary gland (HPM), and hypothalamus–pituitary–skeletal muscle (HPS), as calculated by CellChat. (d) Scatter plots showing the relationship between outgoing and incoming interaction strengths across cell types within each tissue axis. (e–g) Contribution of individual ligand–receptor pairs to UNC5 (e), SLIT (f), and BMP (g) signaling as inferred by CellChat. (h) Dot plot showing scaled expression (color) and percentage of expressing cells (dot size) for key ligand–receptor genes across relevant cell types in the HPO axis, supporting their inferred signaling roles.


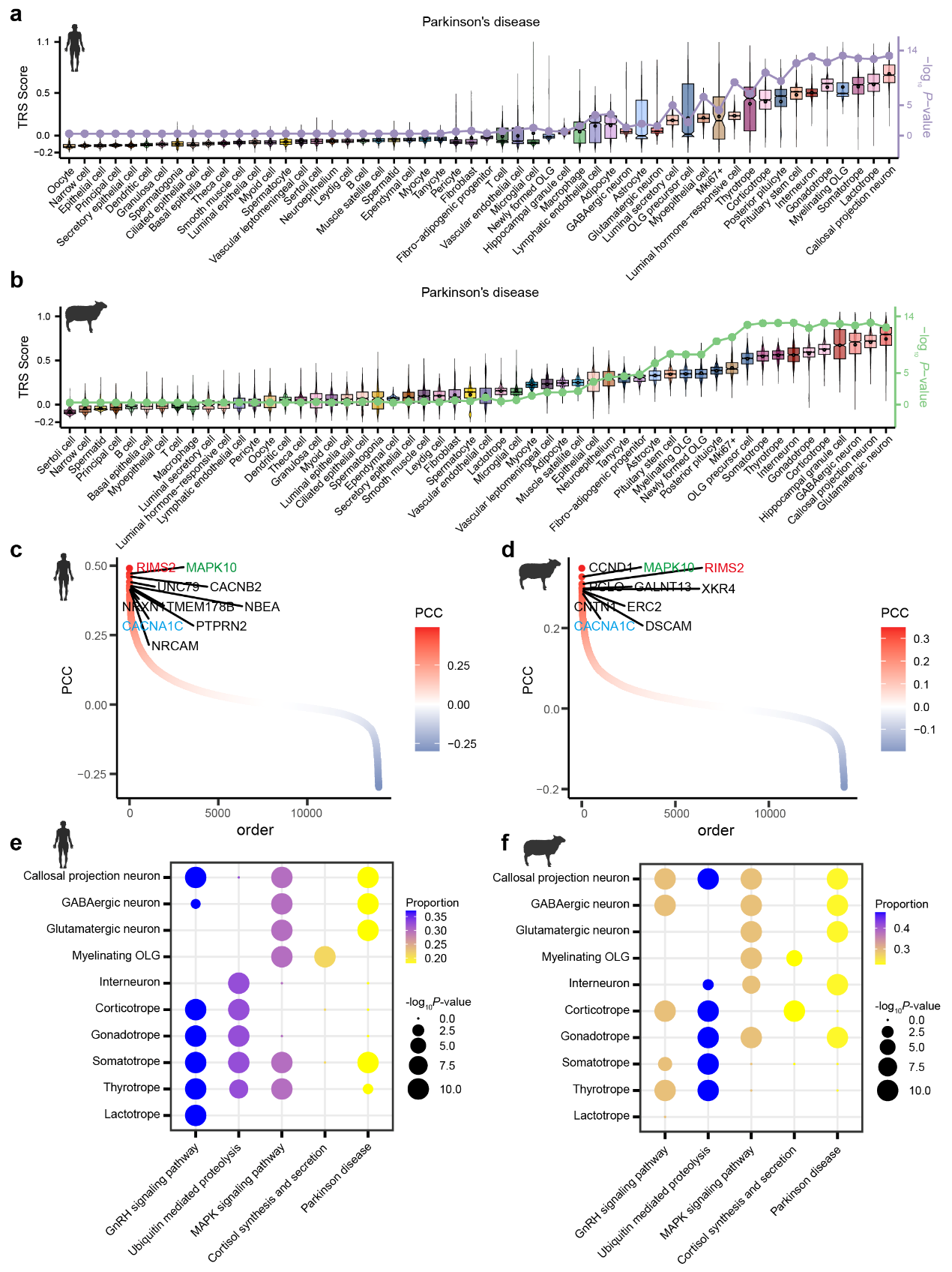


**Figure S24 Conserved cellular and molecular signatures underlying Parkinson’s disease across species.** (a, b) Violin plots of trait-relevant scores (TRS) for each cell type associated with Parkinson’s disease in humans (a) and sheep (b). Dots indicate median TRS values, and lines indicate the significance levels (-log_10_*P*-value) of associations. (c, d**)** Top-ranked trait-relevant genes for Parkinson’s disease in humans (c) and sheep (d), ranked by Pearson correlation coefficients (PCCs) from scPagwas analysis across all single cells. (e, f) Dot plots of conserved trait-relevant pathways associated with Parkinson’s disease in humans (e) and sheep (f) identified by scPagwas. Dot size represents the significant level (-log_10_*P*-value) for each pathway, and color intensity indicates the proportion of cells within each type influenced by these pathways (pathway-level coefficient β > 0).
